# Testing the feasibility, acceptability, and preliminary effect of a novel deliberate practice intervention to reduce diagnostic error in trauma triage: a study protocol for a randomized pilot trial

**DOI:** 10.1186/s40814-022-01212-y

**Published:** 2022-12-12

**Authors:** Deepika Mohan, Jonathan Elmer, Robert M. Arnold, Raquel M. Forsythe, Baruch Fischhoff, Kimberly Rak, Jacqueline L. Barnes, Douglas B. White

**Affiliations:** 1 Department of Surgery, University of Pittsburgh School of Medicine, Pittsburgh, PA USA; 2 Department of Critical Care Medicine, University of Pittsburgh School of Medicine, Pittsburgh, PA USA; 3 Department of Emergency Medicine, University of Pittsburgh School of Medicine, Pittsburgh, PA USA; 4 Department of Neurology, University of Pittsburgh School of Medicine, Pittsburgh, PA USA; 5 Department of Medicine, Division of Palliative Care, University of Pittsburgh School of Medicine, Pittsburgh, PA USA; 6 Department of Engineering and Environmental Policy, Carnegie Mellon University, Pittsburgh, PA USA

**Keywords:** Trauma triage, Clinical practice guidelines, Physicians, Heuristics, Deliberate practice, Diagnostic skill

## Abstract

**Background:**

Non-compliance with clinical practice guidelines in trauma remains common, in part because physicians make diagnostic errors when triaging injured patients. Deliberate practice, purposeful participation in a training task under the oversight of a coach, effectively changes behavior in procedural domains of medicine but has rarely been used to improve diagnostic skill. We plan a pilot parallel randomized trial to test the feasibility, acceptability, and preliminary effect of a novel deliberate practice intervention to reduce physician diagnostic errors in trauma triage.

**Methods:**

We will randomize a national convenience sample of physicians who work at non-trauma centers (*n* = 60) in a 1:1 ratio to a deliberate practice intervention or to a passive control. We will use a customized, theory-based serious video game as the basis of our training task, selected based on its behavior change techniques and game mechanics, along with a coaching manual to standardize the fidelity of the intervention delivery. The intervention consists of three 30-min sessions with content experts (coaches), conducted remotely, during which physicians (trainees) play the game and receive feedback on their diagnostic processes. We will assess (a) the fidelity with which the intervention is delivered by reviewing video recordings of the coaching sessions; (b) the acceptability of the intervention through surveys and semi-structured interviews, and (c) the effect of the intervention by comparing the performance of trainees and a control group of physicians on a validated virtual simulation. We hypothesize that trainees will make ≥ 25% fewer diagnostic errors on the simulation than control physicians, a large effect size. We additionally hypothesize that ≥ 90% of trainees will receive their intervention as planned.

**Conclusions:**

The results of the trial will inform the decision to proceed with a future hybrid effectiveness-implementation trial of the intervention. It will also provide a deeper understanding of the challenges of using deliberate practice to modify the diagnostic skill of physicians.

**Trial registration:**

Clinical trials.gov (NCT05168579); 23 December 2021.

**Supplementary Information:**

The online version contains supplementary material available at 10.1186/s40814-022-01212-y.

## Contributions to the literature

- Improving adherence to trauma triage clinical practice guidelines has the potential to improve the care provided to the 1 million injured patients who present each year to non-trauma centers in the USA.
- Existing methods of increasing adherence to guidelines in trauma have had limited success.
- We describe the development of a novel deliberate practice intervention to reduce diagnostic errors in trauma triage.
- This work contributes to the literature by providing a new method of fostering the use of clinical practice guidelines and will enhance understanding of health professionals’ diagnostic processes.

## Background

A guiding principle in trauma care is that severely injured patients should receive treatment at trauma centers (highly resourced, accredited hospitals) to reduce preventable morbidity and mortality, while minimally injured patients should receive treatment at non-trauma centers to minimize the costs of care [1–3]. Professional organizations have published well-validated guidelines that specify criteria for triage—the categorization of patients as having minor or severe injuries—with the objective of minimizing both under- and over-triage [4]. Despite 40 years of efforts to disseminate the clinical practice guidelines, non-compliance remains common [5–8].

In a series of experimental and observational studies, we identified diagnostic errors as a major cause of non-compliance at non-trauma centers [9, 10]. We found these errors occurred in part because physicians relied on heuristics (pattern recognition or intuitive judgments) to screen patients [11]. In other words, physicians reduced a complex question (“does this patient meet the criteria for transfer to a trauma center?”) to a simpler one (“is this patient badly injured or not?”) [12]. However, features considered pathognomonic for severe injuries occurred infrequently and did not capture the nuances of clinical practice guidelines [13]. Consequently, decisions based on responses to the simpler question resulted in predictable errors in judgment. Few interventions exist that improve the diagnostic skills of practicing physicians.

## Intervention conceptual model

In prior work, we collaborated with Schell Games (Pittsburgh, PA) to develop two serious video games—video games used for applied purposes—to reduce diagnostic errors in trauma triage. In clinical trials, we found that exposure to the games had a small to moderate effect, reducing under-triage by 10–18% [14, 15]. Consistent with best practice guidelines for the development of behavioral interventions, we decided to further refine the interventions to maximize their efficacy before proceeding with widespread distribution [16]. Deliberate practice, defined as goal-oriented, coach-supervised training, has facilitated the acquisition of expertise in domains as disparate as aviation combat and chess [17–19]. The method requires that coaches observe performance on a representative task, identify opportunities for improvement, and provide timely, specific feedback that the learner can use to refine their behavior [19]. As described by Ericsson et al., the efficacy of deliberate practice depends on three variables [17]. First, deliberate practice requires the identification of a representative training task, defined as one that captures the essence of expertise in the domain, and that allows trainees to practice their skills in a consistent and reproducible manner. Ideally, training tasks align challenge with skill, increasing in difficulty as performance improves. Additionally, they should allow for distributed practice, with training spaced across time so that knowledge can transfer from working to long-term memory. Second, deliberate practice entails the delivery of immediate, high-quality feedback to allow the trainee to acquire and to refine the skills necessary to improve their performance on the training task. The coach should provide task-specific, concise feedback, recommending precise actions for behavior change. Third, deliberate practice benefits from the creation of a collaborative relationship between the coach and trainee that fosters autonomous motivation (the desire to perform a task because it generates innate satisfaction or aligns with deeply held values) in the trainee. The development of rapport increases the likelihood that the trainee will persist in practice, remain open to challenging new experiences, and participate in self-reflective processes.

In procedural domains in medicine, a recent systematic review showed a strong correlation between the use of deliberate practice and positive educational outcomes (*r* = 0.71) [20]. However, diagnosis involves cognitive processes that occur unconsciously, making them difficult to observe and to analyze [21]. This may explain why use of deliberate practice to influence diagnostic skill occurs much less frequently [22]. We hypothesized that a deliberate practice intervention might improve diagnostic skill in trauma triage, provided we could develop an appropriate training task where trainees could practice making diagnoses and where coaches could observe their process and provide useful, actionable advice on how to improve their performance. Through an iterative process, our multi-disciplinary team with expertise in adult education, behavioral science, deliberate practice, qualitative research methods, emergency medicine, and trauma surgery developed a deliberate practice intervention to improve the diagnostic skill of emergency medicine physicians working at non-trauma centers by tackling each of these variables in turn.

## Aims and hypotheses

The aims of this study are to test the feasibility, acceptability, and preliminary effect of the novel intervention in a pilot trial. We hypothesize that physicians exposed to the intervention will under-triage ≥ 25% fewer patients than physicians in the control arm (primary outcome). We further hypothesize that ≥ 90% of trainees will receive their intervention as planned (secondary outcome).

## Methods

### Trial design

This study will adhere to the CONSORT guidelines (extension for pilot and feasibility trials) for reporting clinical trials (see Additional file 1). To evaluate the deliberate practice intervention, we will recruit a convenience sample of emergency physicians and randomize them in a 1:1 ratio to the intervention or to a passive control group. Members of the intervention group (‘trainees’) will be paired with a content expert (a ‘coach’), and will receive three, weekly, 30-min remote coaching sessions during which they play a customized, theory-based video game on Zoom and receive feedback on the diagnostic processes they use to triage trauma patients. We will structure the process evaluation using Proctor's Framework for Outcomes in Implementation Research [23]. We will record the coaching sessions and will review the recordings to assess the feasibility and the fidelity of intervention delivery. We will assess the acceptability, adoption, and appropriateness of the intervention through surveys and semi-structured interviews. Finally, we will compare the performance of participants in the intervention and control group on a validated virtual simulation, using under-triage (the proportion of severely injured patients not transferred to a trauma center) as an interim measure of efficacy [3].

### Trial participants

#### Coaches

Three members of the study team with content expertise in trauma (DM, RF) and emergency medicine (JE) will act as the coaches, guiding trainees through the experience of playing the video game, providing instruction on how to diagnose severely injured patients, and reinforcing best practice triage principles. Before the start of the trial, coaches will receive three 1-h training sessions to learn game mechanics, to review the coaching manual, and to gain experience in the use of pedagogical strategies that foster adult learning. We will invite five local emergency medicine residents and advance practice providers who staff emergency departments to participate in these practice coaching sessions. Members of the team with expertise in adult education (RA), deliberate practice (DW), and behavioral science (BF) will observe the sessions and will provide feedback to the coaches on their performance.

#### Trainees

Using a strategy that has proven successful in the past, we will recruit physicians to serve as trainees through respondent-driven sampling. We will contact physicians who have participated in our research previously (*N* ~ 600) and will ask them to refer us to two colleagues who might be willing to participate in the study. Eligible physicians must treat adult patients in the Emergency Department of either a non-trauma center or a Level III/IV trauma center in the USA. We will obtain digital consent from eligible physicians, informing them that the study focuses on evaluating how best to disseminate clinical practice guidelines in trauma. At the time they provide consent, they will also complete a questionnaire describing their personal characteristics.

### Randomization and blinding

A member of the study team (DM) will assign eligible physicians to intervention or control group in a 1:1 ratio, using a randomization schema built using block sizes of 4. After enrolling a participant, she will obtain the intervention assignment from a central database and will inform participants of their assignment. Although we cannot blind study personnel and participants to the intervention after allocation, we will mask condition assignment during the analysis phase.

## Study protocol

After randomization, participating physicians will receive written instructions on how to complete study tasks. We will ask those in the intervention group to select one of the two 3-week blocks of coaching sessions, and will mail them an iPad with the video game and the Zoom app pre-loaded. We will pair trainees with a coach (DM, JE, RF) and will ask coach-trainee dyads to schedule three 30-min weekly sessions during their selected block. At the completion of the 3 weeks, we will ask trainees to participate in a semi-structured, debriefing interview to assess the acceptability of the intervention and to use an online virtual simulation to assess the effect of the intervention on diagnostic errors in triage. We will ask passive controls to complete the same simulation within 3 weeks of enrollment. Study tasks will take approximately 3 h for those in the intervention group and 1 h for those in the control group. Participants will receive personalized reminder emails at weekly intervals for the duration of the study, or until they complete their tasks.

We will use a financial incentive to increase response rates, setting the size using a wage-based model of reimbursement. Physicians in the intervention group will keep the iPad as an honorarium (approximate value $300), while those in the control group will receive a $100 gift card conditional on the completion of the simulation.

### Deliberate practice intervention

The deliberate practice intervention consists of three, weekly, 30-min coaching sessions, conducted remotely. The trainee will play a serious video game (i.e., the training task) on their iPad, sharing their screen through the Zoom app with the coach. Coaches will use the coaching manual to structure these sessions and to ensure the standardization of content. Specifically, the coaches will observe trainees' performance as they play the game, and will provide feedback on the process they use to make diagnoses in trauma triage. The objective of the training sessions is to refine physicians' pattern recognition of severely injured trauma patients (i.e., their heuristics).

#### Training task

*Shift with Friends*, developed in collaboration with Schell Games (Pittsburgh, PA), has 10 levels, each with a 5-step game loop: players triage 10 injured patients over 90 s, compare two of the cases to identify similarities/differences so that they can derive the ‘rule’ for the level, receive standardized feedback on their performance, have the option of triaging an additional 10 cases over 90 s, and finally review the decision principle. The game is grounded in the method of analogical encoding—the idea that the process of performing structured case comparisons allows players to derive decision principles for themselves and therefore makes those decision principles memorable [24]. The game uses five behavior change techniques (repetition and substitution, shaping of knowledge, feedback and monitoring, goal setting, and provision of observable samples of behavior) and delivers them using game mechanics that include time-pressure, variation in the difficulty of different levels, and drag-and-drop mechanics (see Table 1) [25, 26]. The behavior change techniques and game mechanics make the diagnostic task explicit, allowing the coach to observe the trainee’s train-of-thought and to identify precise opportunities for improvement. The graded difficulty of the different levels allows the coach to titrate the complexity of the training task to ensure that novices do not become overwhelmed, while more expert physicians do not become bored. The 5-step game loop, which occurs over the course of 5 to 6 min, facilitates repeated practice sessions. Finally, the game includes standardized feedback, which provides opportunities for a coach to offer additional, personalized comments tailored to the trainee's specific goals.

**Table 1:** Description of deliberate practice intervention (*Shift with Friends*)

Method of behavior change	Behavior change techniques	Deliberate practice intervention	
		Game components (training task)	Coaching components
*Analogical encoding* Described in the organizational sciences literature as a means of recalibrating heuristics by helping novices/trainees to derive memorable, generalizable decision principles for themselves through structured case comparison.	*Repetition and substitution* Practice of the behavior to increase habit and skill. Tasks progress in difficulty over time (grading of tasks).	*Triage*	
		Trainee asked to triage 10 cases over 90 s. ▪ 5 of the cases conform to one decision principle (e.g., penetrating injury). ▪ Each level demonstrates a different decision principle ▪ Trainee can opt to triage another set of 10 cases after reviewing the case comparison step	Coach selects the levels seen by the trainee, curating the user experience based on their experience and knowledge.
	*Shaping knowledge* Advice provided on how to perform the behavior. Information provided about antecedents that reliably predict performance of the behavior	*Case comparison*	
		Trainee asked to compare 2 cases from set of 10 seen during the triage step and asked to identify relevant contextual cues by focusing on similarities and differences between the cases.	Coach guides the identification of relevant contextual cues by using standardized question prompts, and guiding attention to important information.
	*Feedback and monitoring* Monitor and provide information or evaluative feedback on performance of behavior or outcome.	*Feedback*	
		In-game character provides standardized feedback based on trainee performance during the case comparison step.	Coach provides personalized feedback based on player performance during triage step and during the case comparison step.
	*Goal setting* Set or agree on a goal defined in terms of the behavior to be achieved	*Goals*	
		N/A	Coach elicits goals for training session and links to information being covered.
	*Comparison of behavior* Provide an observable sample of the behavior.	*Exemplar*	
		N/A	Coach triages set of 10 cases to demonstrate how use of appropriate contextual cues can facilitate decision making.

#### Coaching manual

To maximize the fidelity of the intervention delivery, we developed a manual to serve as a guide for coaches. The manual specifies the decision principles and the structure of each session [27]. For example, in the first session, participants will cover the decision principle that they should consider the number of body regions involved in the injury when making a triage decision: individually minor or moderate injuries could cumulatively sum to a severe injury complex. We include sample scripts and prompts for coaches to use as they progress across the sessions [27]. The scripts range from suggested language about how to establish rapport (e.g., enlisting them as partners in the endeavor to improve patient outcomes) to recommendations about how to customize feedback based on the trainee’s goals (e.g., “you mention that you struggle to communicate with surgeons at the referral center, here’s what you might say when asking if you can transfer your patient”). The prompts include phrasing for questions, structured so that they progress from perception (e.g., “what do you see here?”) to knowledge building (e.g., “what does this mean for our decision principle?”) to checking for understanding (e.g., “what might happen if you were not able to transfer the patient?”), and probes to elicit thought processes (“tell me more about that?”) [27]. Finally, the coaching manual includes technical vocabulary that coaches can use as a reference and a detailed description of each level of the game with exemplars that covered content, bugs, and predictable errors that might occur during game play by trainees [27].

**Fig. 1:**
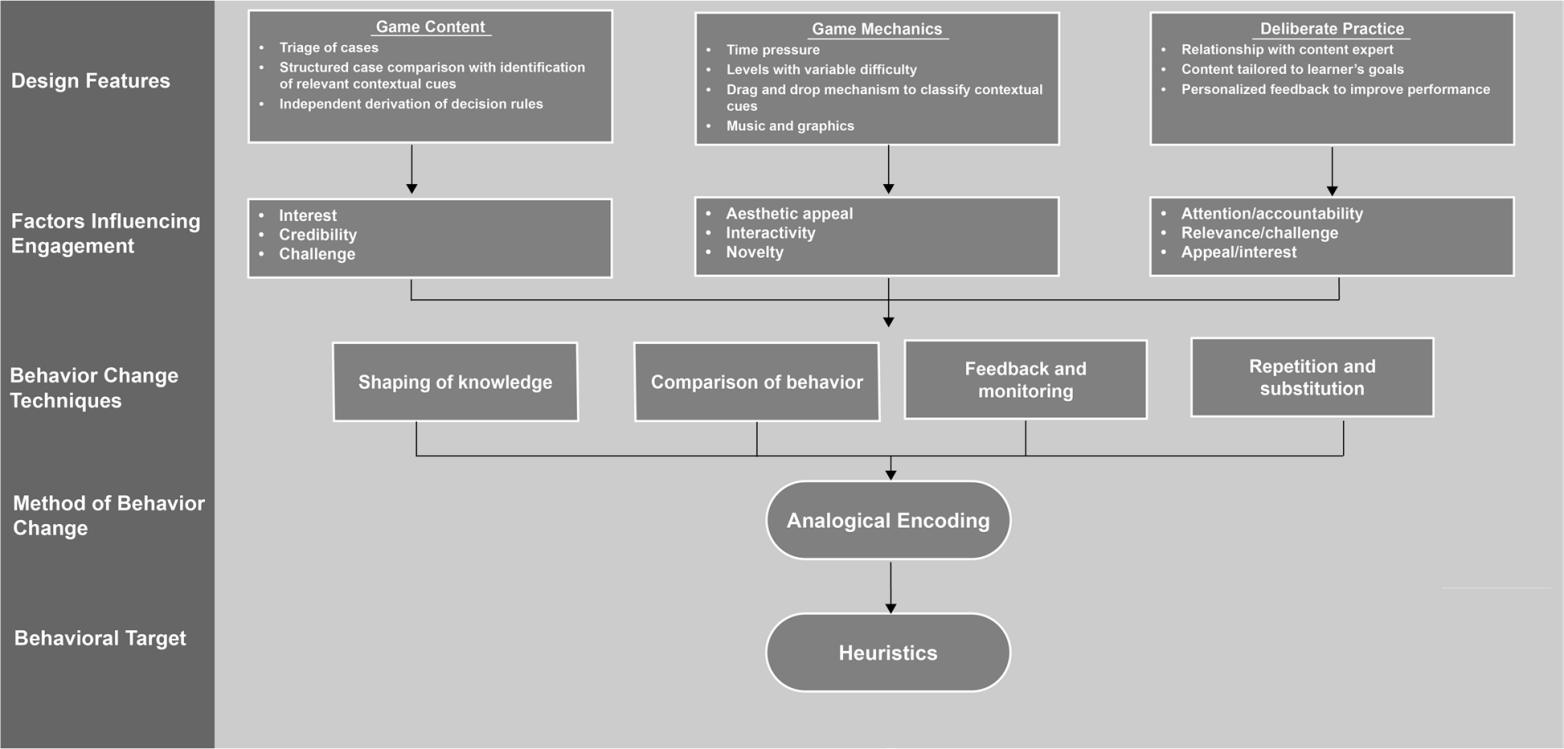
Framework depicting the process of intervention development. We attempt to make transparent how each component of the intervention is intended to intervene on the behavioral process, with relationships among phases depicted with arrows

**Fig. 2:**
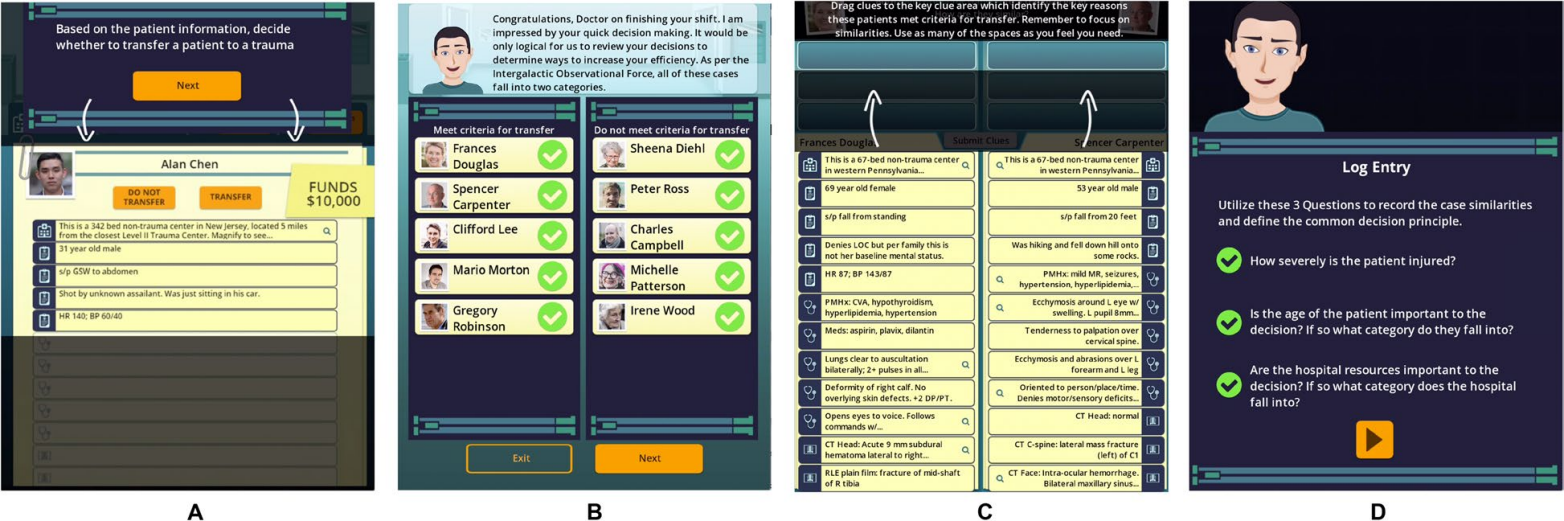
Screenshots from *Shift with Friends* demonstrating the steps in the game loop from level 1. **a** triage of 10 cases in 90 s. **b** generic feedback provided by in-game character on performance during triage round. **c** Review of contextual cues to identify generalizable principles. **d** Generation of summative decision principle. Game play is supplemented by interactions with the coach to ensure that content is tailored to the trainee’s goals and that feedback is personalized to their performance

We refined the coaching manual iteratively based on observations made during a series of practice coaching sessions, introducing additional pedagogical strategies to improve coaching performance (see Table 2) [28]. For example, coaches could not always quickly parse the etiology of errors made during game play, impeding their ability to provide relevant, concise feedback to trainees. We therefore introduced a think-aloud technique, asking trainees to articulate the trajectory of their thoughts as they triaged patients or compared cases. We will report further adaptations to the intervention during the pilot trial using the FRAME (Framework for Reporting Adaptation and Modifications–Expanded) checklist [29]. We provide a schematic of the components of the intervention in Fig. 1, a logic model of the intervention in Additional file 2, screenshots of the game (*Shift with Friends*) in Fig. 2, and a current draft of the coaching manual in Additional file 3.

**Table 2:** Key pedagogical strategies emphasized in the coaching manual. We iteratively refined the coaching manual to specify relevant pedagogical principles based on observations during practice coaching sessions [26]

Pedagogical strategy	Description of strategy	Rationale	Iteration
Planning for error	A strategy of anticipating predictable mistakes made by trainees, and preparing a response in advance. Preparation not only increases the rate of recognition of these errors but also increases the likelihood of a productive response.	Improve the quality of **feedback**	Initial draft
Questioning	A strategy for phrasing questions so that they begin with perception (“what do you see?”) and then move to knowledge building (“what does it mean?”) and finally to checking for understanding (“what is our objective here?”).	Improve the quality of the **training task**	Initial draft
Exemplar planning	A lesson plan with correct answers to the questions that the coach will ask. If agreement on the response is established before the start of the coaching session, it reduces variability in execution and preserves autonomy.	Improve the quality of **feedback**	Initial draft
Creating a culture of error	A strategy for encouraging trainees to think of the 'wrong' answer as the first, positive, and critical step toward getting it 'right, socializing them to acknowledge and to share mistakes with interest and fascination.	Promote autonomous **motivation**	Introduced after first practice session
Active observation	A strategy of prioritizing the recognition of specific errors that commonly compromise performance, by actively tracking their occurrence. This strategy improves analysis of errors of judgment and can improve the quality of the feedback provided to the trainee.	Improve the quality of **feedback**	Introduced after second practice session

## Data sources and management

We summarize the timeline, study procedures, and the data collection plan in Table 3.

**Table 3:** Summary of timeline, study procedures, and data collection

Procedures	Recruitment/randomization	Enrollment	Weeks 1–3	Post-intervention
	▪ Recruitment email sent to national sample of emergency medicine physicians. ▪ Respondents randomized to intervention or control groups.	▪ Instructions sent to all participants ▪ Trainees sent iPads with game loaded	▪ 30 min weekly game-play occurs at convenience of coach-trainee dyad; sessions occur by Zoom; interaction video-taped ▪ Reminder email sent to control physicians^a^	▪ Debriefing interview scheduled ▪ All participants receive reminder emails about completion of simulation^a^ ▪ Control physicians sent conditional honorarium
**Data collection**				
Intervention		Personal characteristics questionnaire	Assess intervention with the User Engagement Scale–Short Form after week 3 session	▪ Simulation ▪ Debriefing semi-structured interview
Control		Personal characteristics questionnaire		▪ Simulation

^a^As necessary

### Questionnaire to assess personal characteristics

At the time of enrollment, each physician participant will answer questions about age, sex, race, ethnicity, educational background (board certification, ATLS certification, years since completion of residency), and practice environment (trauma designation of their hospital).

### Tracking database

We will maintain a tracking database with a list of scheduled coaching sessions. One member of the study team (DM) will update the database daily with the status of sessions (completed v. not, videotaped v. not), which we will use to assess the feasibility of delivering the intervention.

### Coaching sessions

All the coaching sessions will be recorded and uploaded to a secure server. Two coders (KJR, JLB) will review the recordings, applying the coaching manual to assess the fidelity of intervention delivery, and the Wisconsin Surgical Coaching Rubric to evaluate the performance of the coaches. The Rubric assesses four domains, asking if the coach (1) engages the trainee as an equal participant in learning; (2) uses questions and prompts to guide trainee in self-reflection; (3) provides constructive feedback; (4) guides goal setting [30].

### Post-intervention debriefing materials

After completing the coaching sessions, we will ask participants in the intervention group to complete the User Engagement Scale–Short Form to assess their engagement with the intervention [31]. The scale has 12 items that measure focused attention, perceived usability, aesthetic appeal, and reward. Additionally, two qualitative researchers (KJR, JLB) will conduct 20-min semi-structured interviews with trainees, probing their opinion the acceptability, adoption, and appropriateness of the intervention, inviting suggestions for change, and asking their opinion about the quality of the coaching.

### Virtual simulation to assess effect size

Trial participants will log into a website to complete a virtual simulation designed assess physician decision making in trauma triage. We previously collaborated with a gaming company (Breakaway Ltd.; Hunt Valley, MD) to develop a 2D simulation to reflect the environment of an Emergency Department at a non-trauma center. The simulation has both internal reliability as well as criterion validity. Importantly, we have found that, at the group level, physicians make similar decisions for trauma patients on the simulation as they do in real-life [11]. Responses to the virtual simulation will be transmitted from the website to a secure server hosted by the University of Pittsburgh.

The simulation includes ten cases: four severely injured patients, two minimally injured patients, and four critically ill non-trauma cases. Users must evaluate and manage these cases over 42 min, simulating a busy ED shift. New patients arrive at pre-specified (but unpredictable) intervals, so that physicians must manage multiple patients concurrently. Each case includes a 2D rendering of the patient, a chief complaint, vital signs which update every 30 s, a history, and a written description of the physical exam. Without appropriate clinical intervention by the player, severely injured patients and critically ill distractor patients decompensate and die over the course of the simulation.

Physicians manage patients by selecting from a pre-specified list of 250 medications, studies, and procedures. Some orders affect patients’ clinical status, leading to corresponding changes in their vital signs and physical exam. Other orders generate additional information, presented as reports added to the patients’ charts. Each case ends when the player either makes a disposition decision (admit, discharge, transfer) or the patient dies.

## Analyses

We will include in the implementation outcome analysis physicians who do not complete all three coaching sessions (i.e., intention-to-treat). We will exclude from the service outcome analysis those who do not complete the simulation (i.e., those who have missing data). We will summarize physician characteristics using means (standard deviations) for continuous variables and proportions (%) for categorical variables.

### Feasibility, fidelity, acceptability, adoption, and appropriateness (implementation outcomes)

We will use Proctor’s framework to define the implementation outcomes for the process evaluation [23]. We will define ‘feasibility’ as the practicability of delivering this intervention as planned, and will quantify the proportion of coach-trainee dyads that complete all three 30-min training sessions. We will define ‘fidelity’ as adherence to the coaching manual and will quantify the number of session components provided to each participant. As a secondary measure of this construct, we will compare differences in the quality of coaching across domains of the Wisconsin Surgical Coaching Rubric. We will define ‘acceptability’ as the perception that a given intervention is agreeable, palatable, or satisfactory, ‘adoption’ as the intention to try the intervention, and ‘appropriateness’ as the perceived fit of the intervention. To assess these constructs, we will summarize responses to the User Engagement Scale-Short Form, and will code the semi-structured interviews, categorizing responses to the range of questions about the acceptability (e.g., “did the experience meet your expectations?”), adoption (e.g., “have you been able to use any of the information provided in the sessions?”), appropriateness of the intervention (e.g., “how appropriate was the intervention for you?”), suggestions about how to improve the experience (e.g., “do you have any suggestions to increase the feasibility of implementation?”), and the quality of the coaching (e.g., “what do you think the coach added to the experience?”).

### Efficacy (service outcome)

For the purposes of this analysis, we will define ‘efficacy’ as compliance with clinical practice guidelines in the triage of trauma patients and will concentrate on disposition decisions made during the simulation. We will score the decisions for each severely injured trauma case based on American College of Surgeons guidelines as triaged appropriately or not [3]. We will summarize triage decisions at the group-level and calculate the proportion of under-triage by group:

$$\frac{\textrm{number}\ \textrm{of}\ \textrm{severely}\ \textrm{injured}\ \textrm{patients}\ \textrm{not}\ \textrm{transferred}\ \textrm{to}\ \textrm{trauma}\ \textrm{centers}}{\textrm{total}\ \textrm{number}\ \textrm{of}\ \textrm{severely}\ \textrm{injured}\ \textrm{patients}}$$

We will assess the effect of the intervention compared with the control on trainee performance using generalized linear models, clustered at the trainee level.

### Decision to proceed with a definitive trial of the intervention

Results from the analysis will inform the decision about whether to proceed with a future definitive trial of the intervention. Given the complexity of the intervention, we will classify the pilot trial as a success if the intervention has a large effect (i.e., exceeding that of the video games alone) and if ≥90% of trainees receive the intervention as planned.

## Human subjects and power calculation

We used Cohen’s method of estimating power for behavioral trials, basing our calculation on the assumption the data will be continuously and normally distributed [32]. We have designed the experiment to detect a 25% (large effect size) reduction in under-triage between physicians in the intervention and control groups, with an alpha of 0.05 and a power of 80%. Based on these estimates and anticipating a 67% retention rate in the control arm, we plan to recruit 60 physicians (30 physicians per group).

## Security, ethics, and dissemination

### Data security

On enrollment in the trial, participants will receive a unique identifier. All participants will use that identifier to login to the website that hosts the virtual simulation. Those assigned to the deliberate practice group will use it to access *Shift with Friends* as well. Only the primary investigator and the qualitative researchers will have access to the linkage file connecting the identifier to the physician’s name and contact information. This file will be encrypted and stored on a secure server at the University of Pittsburgh.

### Ethics

The University of Pittsburgh Institutional Review Board approved this study (STUDY20120026). We do not plan any interim analyses. We will ask participants to communicate any adverse events or unintended effects of participation via email. We have registered the trial on clinicaltrials.gov (NCT05168579).

### Dissemination of results

Results from the study will be reported to the public through manuscripts and oral presentations at national meetings. We will provide an abstract of the findings to all participants. Access to the de-identified dataset will be made available upon written request to the study team.

## Discussion

This paper summarizes the protocol we will use to test the feasibility of using a deliberate practice intervention to improve physician diagnostic skill in trauma triage. Our overarching aim is to increase physician adherence to clinical practice guidelines at non-trauma centers. Strengths of the intervention include an explicit grounding in theory, translation of deliberate practice to the refractory problem of physician diagnostic skill, and an iterative, user-centered design process focused on ensuring the fidelity of intervention delivery.

Few interventions exist to improve the diagnostic skill of physicians who have completed graduate medical education. The gold standard in trauma triage is *Advanced Trauma Life Support* [33]. Designed by the American College of Surgeons, the textbook-based course exposes learners to rule-based algorithms and essential skills over 16 h. Implicitly, ATLS uses the rational actor model of decision making as its theory of behavior, and attempts to shape knowledge as its behavior change technique [34, 21]. Over 1 million providers have received their ATLS certification; yet surprisingly little evidence exists that certification changes performance in practice [33, 35]. Based on formative research, we believe that the dual process model of cognition better explains diagnostic skill in trauma triage and have experimented with different methods of behavior change aligned with this theory to improve triage practices [12, 36]. Prior interventions have had small to moderate effect sizes [14, 15, 37]. Consequently, in response to best-practice guidelines for the development of behavioral interventions, we will now test deliberate practice as an alternative method of behavior change to ensure that we have maximized the efficacy of our interventions before proceeding to widespread distribution [16].

Deliberate practice is an appealing adjunct because of successes in other domains (e.g., music, sports, combat) and theoretical compatibility with the dual process model of cognition underlying our video games [17–19]. However, its application to influence diagnostic skill has occurred infrequently, perhaps because of the difficulty of designing appropriate training tasks [22]. Diagnosis routinely occurs under complex task conditions, difficult to replicate in the laboratory or classroom. Patients rarely present with pathognomonic features. Physicians must make decisions rapidly and while distracted by competing demands on their attention [38]. Moreover, diagnosis occurs unconsciously [39]. Consequently, coaches may struggle to understand the source of errors and to provide useful, actionable feedback. To address these challenges, we selected a puzzle video game, where players must triage 10 patients over 90 s, as the basis of our training task, combined with a think-aloud approach to allow the coach insight into the thought processes of the trainee. If successful, this approach offers a potential template for others interested in using deliberate practice to improve diagnostic skill.

Our intervention and protocol development focused on the importance of ensuring the fidelity of intervention delivery, as recommended by the NIH's Science of Behavior Commission [40]. The efficacy of deliberate practice depends on the ability of the coach to provide personalized, relevant feedback and to foster a collaborative relationship with the trainee that motivates him/her to engage with learning. Coaches need a wide variety of complex skills to accomplish these tasks; lack of the skills can compromise the trainee experience, and can have a negative effect on behavioral outcomes [41]. We developed a coaching manual with sample scripts, question prompts, and didactic information to guide coaches as they delivered the content of the intervention. We iteratively refined the manual based on observations made during practice and pilot coaching sessions, specifying pedagogical principles that coaches should use to address predictable difficulties that arose as users engaged with the material [28]. We anticipate identifying additional opportunities to improve our protocol for delivering the intervention during this pilot trial.

The study has several potential limitations. First, we will use a convenience sample to test the efficacy of the interventions, which may not represent the general population of physicians who serve in non-trauma emergency departments. Second, we use a virtual simulation with a limited number of cases to assess outcome rather than decisions made in practice. Our previous validation study provides evidence of the simulation’s ability to predict group-level performance in practice, making it, we believe, a reasonable interim outcome measure. If the present study affirms the potential of such interventions, real-world efficacy and effectiveness testing would be warranted. Third, the size of the sample precludes the ability to adjust for the influence of individual coach differences on the estimate of the effect of the intervention. We plan qualitative analyses to evaluate the quality of the coaching, which will inform future tests of the intervention. Fourth, we selected deliberate practice as an adjunct to our existing video game as a means of augmenting its efficacy without iteratively testing a full panel of options. This decision had a pragmatic justification: a multiphase optimization strategy approach (arguably the best-practice method of developing effective behavioral interventions) would have exceeded our limited resources [42]. Moreover, we had conceptual reasons to believe that deliberate practice could effectively change behavior. However, our failure to consider a full suite of methods of behavior change may have limited the rigor of the work.

## Conclusions

We developed a novel intervention to improve diagnostic skill in trauma triage using principles adapted from both the dissemination and implementation literature and the literature on the acquisition of expertise. We will test the fidelity, acceptability, and efficacy of the intervention in a pilot feasibility trial, which will allow us to understand the success of our theoretical behavioral and design principles.

## Supplementary Information

**Additional file 1.** CONSORT 2010 checklist of information to include when reporting a pilot or feasibility trial*.

**Additional file 2.** Logic diagram of the intervention and its anticipated mechanism of action. We show the outcomes that we will not assess during this pilot trial with an asterisk.

**Additional file 3.** Coaching Manual for Shift with Friends (version 6).

## Data Availability

*Shift with Friends* is available for download on the iOS Apple Store. A de-identified dataset will be made available upon request to the PI, and after appropriate authorization by the University of Pittsburgh Office of Research.
