# Supplementary figures and images for: Testing the feasibility, acceptability, and preliminary effect of a novel deliberate practice intervention to reduce diagnostic error in trauma triage: a study protocol for a randomized pilot trial
Source: Pilot Feasibility Stud. 2022 Dec 12;8:253. doi: 10.1186/s40814-022-01212-y (PMC9743730; doi:10.1186/s40814-022-01212-y)

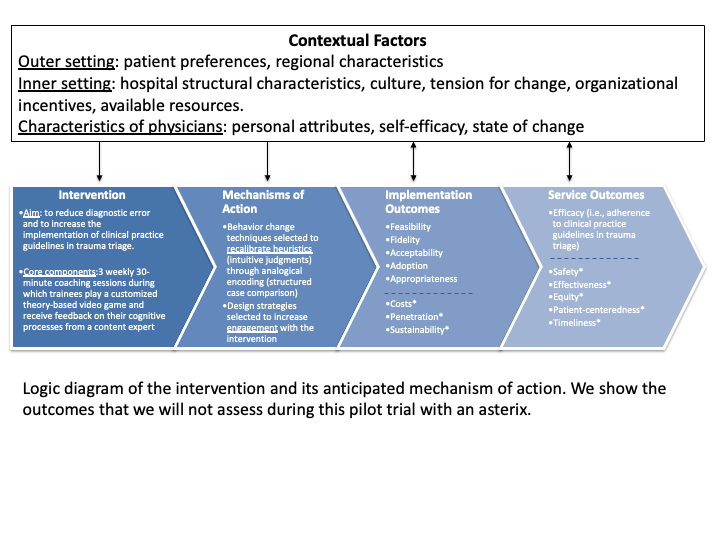

Supplement: Supplementary file 2 — Additional file 2. Logic diagram of the intervention and its anticipated mechanism of action. We show the outcomes that we will not assess during this pilot trial with an asterisk. [file 40814_2022_1212_MOESM2_ESM.tiff]
