# Supplementary material for: Testing the feasibility, acceptability, and preliminary effect of a novel deliberate practice intervention to reduce diagnostic error in trauma triage: a study protocol for a randomized pilot trial
Source: Pilot Feasibility Stud. 2022 Dec 12;8:253. doi: 10.1186/s40814-022-01212-y (PMC9743730; doi:10.1186/s40814-022-01212-y)
Supplement: Supplementary file 3 — Additional file 3. Coaching Manual for Shift with Friends (version 6). [file 40814_2022_1212_MOESM3_ESM.docx]

**Coaching Manual for *Shift with Friends (version 6)***

**Investigative Team:**

Deepika Mohan (PI)

Jonathan Elmer

Raquel Forsythe

Jacqueline Barnes

Kim Rak

Baruch Fischhoff

Bob Arnold

Doug White

**Funding**:

National Institute of Aging R21 AG072072

Table of Contents

[Background 3](#_Toc106952080)

[Overview 3](#_Toc106952081)

[Game Content 4](#_Toc106952082)

[Technical Vocabulary – what the coach should know 5](#_Toc106952083)

[Why should we transfer patients to trauma centers? 5](#_Toc106952084)

[Patient recognition 6](#_Toc106952085)

[Injury Severity – Physiological characteristics 7](#_Toc106952086)

[Injury Severity – Anatomical characteristics 8](#_Toc106952087)

[Patient reserve 9](#_Toc106952088)

[Hospital resources. 9](#_Toc106952089)

[Pedagogical Strategies 10](#_Toc106952090)

[Curriculum 12](#_Toc106952091)

[Overview of structure of coaching sessions 12](#_Toc106952092)

[Structuring the Three Sessions 13](#_Toc106952093)

[Notes to the coaches 13](#_Toc106952094)

[Session 1 13](#_Toc106952095)

[Session 2 16](#_Toc106952096)

[Session 3 17](#_Toc106952097)

[Modelling document 18](#_Toc106952098)

[Overview 18](#_Toc106952099)

[Details of each level 20](#_Toc106952100)

[Level One 20](#_Toc106952101)

[Level Two 21](#_Toc106952102)

[Level Three 22](#_Toc106952103)

[Level Four 23](#_Toc106952104)

[Level Five 24](#_Toc106952105)

[Level Six 25](#_Toc106952106)

[Level Seven 26](#_Toc106952107)

[Level Eight 27](#_Toc106952108)

[Level Nine 28](#_Toc106952109)

[Level Ten 29](#_Toc106952110)

[Selected References 30](#_Toc106952111)

[Consent 36](#_Toc106952112)

# Background

## Overview

Diagnostic error is a particularly important problem for the 1 million patients who present to non-trauma centers every year after trauma. Emergency medicine physicians must rapidly categorize patients as having minor or severe injuries based on limited information, and decide whether or not to transfer the patients to a trauma center (*triage)*. Professional organizations have published well-validated guidelines that specify criteria for categorizing injuries. Despite 40 years of performance improvement initiatives in trauma, under-triage remains common (incidence: ~50%), particularly among patients over 65 (incidence: >80%). Severely injured patients treated at non-trauma centers have worse outcomes than those treated at trauma centers, including a 25% increase in mortality, greater disability at discharge, greater pain at one year, and reduced rates of independent living.

Observations from our group demonstrate that physicians rely on *heuristics* (intuitive judgments) to identify severely injured patients. For example, physicians have a five-fold greater likelihood of transferring patients after a penetrating mechanism of injury (e.g., gunshot) than after a blunt mechanism (e.g., fall). In other words, they reduce a complex question (do patients formally meet the criteria for transfer to a trauma center) to a simpler one (are patients ‘sick’ or ‘not sick’). Unfortunately, pathognomonic features of the 'sick' patient (e.g. hypotension, penetrating injury) are frequently absent: in one study, they occurred in ≤25% of cases.

The absence of an effective method of recalibrating heuristics is a critical barrier to improving patient outcomes after injury. If poorly calibrated, the use of heuristics results in attention to irrelevant contextual cues and produces diagnostic errors. However, when well-calibrated*,* they produce rapid, cognitively frugal solutions to complex problems. Think of Captain Chesley Sullenberger landing US Airways Flight 1549 in the Hudson River after a bird strike rendered both engines inoperable: when asked afterwards how he had made decisions, he replied, "we had to work almost intuitively." Behavioral scientists agree that people develop well-calibrated heuristics when: a) the decisional context provides reliable, valid cues to the problem, and b) the person has the opportunity to learn the relevant contextual cues (see Figure 1). Two major problems threaten efforts to improve heuristics in trauma triage.

**Figure 1. Conceptual model of cognition in trauma triage**. Two sets of processes produce diagnoses: System 1 (heuristic) processes are responsible for the majority of judgments; System 2 (analytic) processes serve as a corrective factor. With sufficient experience, the use of rule based algorithms can become intuitive. In theory, people can also deliberately shift from one system to another, but this rarely occurs in practice.

*Problem 1. Emergency medicine physicians work in unpredictable environments.* Emergency physicians at non-trauma centers evaluate 1000 patients for every 1 with severe injuries.^48^ The rarity of the stimulus means that physicians lack the opportunity to gain the feedback necessary to calibrate their ability to distinguish between patients with minor and severe injuries. Ideally, a situation outside the norm should prompt a deliberate shift to guideline-based algorithms. However, in practice, the complexity of the work environment and the variability of contextual cues make it difficult to recognize when to pause to engage in rule-based decision making.

*Problem 2. Simple exposure to rule-based algorithms does not change behavior*. Heuristics reflect deeply engrained associations. Standard educational efforts – lectures, podcasts, review of journal articles – may improve decision making in the laboratory. However, they have limited effectiveness in real life because of poor task transference.

To address these problems, we are developing *Shift with Friends*, a deliberate practice intervention. The intervention will consist of three thirty-minute coaching sessions, conducted by video-conferencing, between physicians working at non-trauma centers ('trainees') and physicians with experience working at trauma centers ('coaches'). Trainee-coach dyads will meet for 30 minutes/week for 3 weeks, by video-conferencing, to play an existing video game (*Shift: The Next Generation*) and to use it to practice pattern recognition. In the sections that follow, we describe the content of the game, technical information that the coaches should know, pedagogical strategies to be used by coaches, the curriculum for the three coaching sessions, and specific details about nuances of the game.

## Game Content

*Shift: The Next Generation* is a puzzle video game. Players engage in *analogical encoding* – structured case comparison – to derive their own decision principles for triage. Specifically, players review cases and then identify contextual cues associated with the presentation of severely injured patients. Next, they synthesize those cues into simple, unifying triage principles. Theoretically, the process of derivation makes the principles memorable, and therefore more likely to become part of the physicians' heuristics. The game has approximately 2 hours of content, covers 10 triage decision principles, and allows repeated play of selected sections. It should lend itself well to deliberate practice because coaches can observe the contextual cues that physicians highlight during the process of case comparison and can provide personalized feedback on how they should integrate those cues into the pattern that they use when diagnosing trauma patients (i.e. recalibrate their heuristics).

Through a review of the literature and the CDC/ACS-COT guidelines for triage at non-trauma centers, we categorized injured patients into three groups (Table 1).

**Table 1.** Three groups of injured patients who present to non-trauma centers.

| **Must transfer** | **Maybe should transfer – depending on hospital resources** | **Do not transfer** |
| --- | --- | --- |
| 1. Severe injuries 2. Frail patients with serious injuries 3. ≥70-year-old patients with serious injuries 4. Patients with ≥2 serious injuries that involve different body regions | 1. Robust/young patients with serious injuries 2. Frail/old patients with moderate injuries** | 1. Patients with minor injuries 2. Robust/young patients with moderate injuries** |
| Definitions:  *minor* is defined as AIS=1  *moderate* is defined as AIS=2 (e.g., clavicle fracture, 1-2 rib fractures, closed long bone fracture other than femurs)  *serious (or severe, non-life-threatening)* is defined as AIS=3 (e.g., femur fracture, 3+ rib fractures, small SDH)  *severe (or severe, life-threatening)* is defined as AIS=4 (e.g., moderate SDH, injuries causing hemorrhagic shock, paralysis)  *frailty* is defined as the phenotype that includes slowed walking speed, low physical activity, unintentional weight loss, low energy, and low grip strength (presence of 3/5 indicates frailty). | | |

**Principles not covered in the video game.

Next, we distilled this information into ten decision principles for trauma triage, creating one level of game play for each principle (Table 2). We provide a rationale for each decision principle in the section that follows ("Technical Vocabulary").

**Table 2**. List of decision principles encoded into *Shift: The Next Generation*

| **Decision principle – short form** | **Detailed description of the decision principles** |
| --- | --- |
| **Penetrating injury** | Transfer patients with penetrating injuries to the head, torso, or proximal extremities to trauma centers. |
| **Intubation** | Transfer patients who are intubated (either because of respiratory failure or altered mental status) to a trauma center. |
| **Mangled extremity** | Transfer patients with mangled extremities to trauma centers. |
| **Paralysis** | Transfer patients with paralysis or lateralizing neurological signs to trauma centers. |
| **Shock** | Transfer patients with hypotension (SBP <90) to trauma centers. |
| **≥2 systems injured** | Transfer patients with serious injuries that involve more than one body region to trauma centers. |
| **Frail + serious injury** | Transfer frail patients with serious injuries to trauma centers. |
| **>70 + serious injury** | Transfer elderly patients (those with age over 70) with serious injuries to trauma centers. |
| **Young + serious injury + small** | Transfer young patients with serious injuries to trauma centers if the non-trauma center lacks the resources available to manage their care. |
| **Minor injury** | Patients with minor or moderate injuries should not be transferred to trauma centers. |

## Technical Vocabulary – what the coach should know

We provide a summary of the relevant literature that informed the development of *Shift: The Next Generation*. References are provided at the end. This is information that the coach should know, and can use to supplement the information provided within the game. We have highlighted (with a blue border and/or bolded text) a few facts that have seemed particularly useful/interesting to trainees.

### Why should we transfer patients to trauma centers?

- The objective of the game – and of trauma guidelines in general – is to improve physicians' pattern recognition.
- There is robust evidence that trauma centers improve outcomes for severely injured patients.
  - National Study of Costs and Outcomes of Trauma (a prospective, observational cohort study) – adjusted mortality for patients with a major trauma at trauma centers is lower than adjusted mortality for patients with a major trauma treated at non-trauma centers (in-hospital relative risk 0.80, 95% CI 0.66-0.98; 1-year mortality relative risk 0.75, 95% CI 0.60-0.95). The benefit was concentrated among patients with a maximal AIS of 4 or higher. It also appeared that the benefits were larger for patients younger than 55, however the authors note that they had a small number of older patients enrolled in the study.
    - Gold standard paper in trauma outcomes research. Follows a decade of retrospective cohort studies that showed that treatment at trauma center appeared to be protective, after adjusting for injury severity and selection bias.
    - Did not necessarily speak to the benefits of inter-facility triage.
    - Introduced caveats about the benefit of trauma centers for older adults.
  - Patients who experience inter-facility triage rather than remaining at non-trauma centers have a reduction in mortality after adjusting for illness severity (OR 0.67, 95% CI 0.48-0.94 [Newgard et al., 2007]).
  - Retrospective cohort studies suggest that the benefits of trauma centers do extend to those over 65 (or 70 depending on the study). Goodmanson et al. found that trauma center care was associated with a decreased mortality risk compared with non-trauma center care (OR 0.83, 95% CI 0.69-0.99). Matsushima et al., found that an increasing volume of geriatric patients is associated with a reduction in mortality (OR 0.75, 95% CI 0.61-92), major complications (OR 0.79) and failure to rescue after a major complication (OR 0.65). Demetriades et al., found that early activation of the trauma team and aggressive use of ICU care reduced mortality by 30%.
  - Benefits appear to be about more than mortality. For example, a higher proportion of geriatric trauma patients treated at trauma centers are discharged home (64% vs. 73% [Maxwell et al., 2013]). Patients experiencing high-energy lower extremity injuries, and treated at trauma centers, are more likely to report better physical functioning at one year (Mackenzie et al., 2008). Patients treated at trauma centers are more likely (OR 1.2 [blunt]; OR 1.4 [penetrating]) to achieve total independence at one year (Nirula et al., 2006). Patients treated at Level I trauma centers are 23-38% more likely to return to work at 3 months (Prada et al., 2012).
- The effect of the trauma center is likely the product of some combination of: a) access to specialized surgical services (Macias et al., 2009 documented a two-fold increase in the number of surgical interventions for spinal cord injuries at trauma centers compared with non-trauma centers, which in turn translated into a 30% reduction in paralysis at discharge); b) the volume-outcome relationship (Nathens et al., 2001 showed that high volume trauma centers with >650 admissions per year have reductions in mortality and length of stay compared with those trauma centers that have ≤650 admissions); c) multi-disciplinary coordinated care (see Bach et al., 2017).
- HOWEVER, we should/can also acknowledge that transferring patients to trauma centers is not an unmitigated benefit – the reason that the NAM advocates for **regionalization** and not **centralization** (see 2010 IOM report of its workshop on regionalizing emergency care). First, the movement of patients, out of small, community hospitals, erodes their revenue stream and can jeopardize their financial stability with long-term consequences for their viability. Second, the movement of trauma can impact adjacent specialties (e.g., orthopedics) and can have a trickle-down effect on expertise in other domains. Third, the movement of patients out of their communities can impose a hardship on the patients and their families. Finally, it may overwhelm the capacity of trauma centers, contributing to adverse outcomes.

### Patient recognition

The definition of a major trauma patient is a person who has sustained a potentially life- or limb-threatening injury. The objective of triage is to identify major trauma patients – or those with severe injuries.

- There are numerous injury severity scoring systems to identify those with 'major' or 'severe' injuries, some that are calculated in real-time and others that are calculated retrospectively based on trauma registry or administrative data. [See Chawda et al., 2003 for an introduction into different scoring systems.]
- All of the existing scoring systems have their limitations [West et al., 2000]. These include:
  - Failure to discriminate among patients with minor/moderate injuries. Specifically, scores based on physiologic data alone do not discriminate well among patients with different injury severity. For example, the GAP (GCS, Age, Pressure) score [Kondo et al., 2011] categorizes patients into high/medium/low risk groups where high risk = in-hospital mortality ≥50%, medium risk = in-hospital mortality >5%-49%, low risk = in-hospital mortality ≤5%. But the average mortality for most cohorts in the US is 3-10%, making this method of screening patients ineffective.
  - Inability to use the score in real-time. Scores that incorporate anatomic data do a better job of discrimination but cannot be calculated in real-time, thereby limiting their utility as tools for triage.
  - Loss of information. Physiologic and anatomic data are combined into intermediate scores, which are then further combined to achieve a probability of mortality. However, in the process, information is lost.
  - Failure to adjust for moderators of injury severity. Most scores do not address the effects of interactions among injuries, or the influence of care on the perceived severity of an injury (a severe injury at hospital A may not be a severe injury at hospital B).
  - Inadequate calibration. Many of the scores that use administrative or registry data are non-monotonic [see Cook et al., 2014], making interpretation problematic.
- The cases are designed using AIS (Abbreviated Injury Scale) scores, and coaches should promote this vocabulary (if appropriate). It is important for learners to become familiar with the spectrum because triage decisions are conditional on injury severity + patient reserve + hospital resources. AIS scores allow the categorization of injuries as minor, moderate, serious, and severe. The scoring framework was developed in 1971 as a means of standardizing assessment of blunt injuries after motor vehicle collisions, and reflects a consensus assessment of the severity of 1300 different injuries. It has been updated six times since it was first published. It ranges from 1 to 6.
  1. Minor (AIS = 1): contusions, concussion, facial fractures, distal extremity (e.g., hand/feet) injuries.
  2. Moderate (AIS = 2): clavicle fracture, 1-2 ribs, bad facial injuries, closed long bone upper/lower extremity injuries.
  3. Severe, non-threatening (AIS = 3): open long bone fractures, closed femur fractures, small traumatic brain injuries, ≥3 ribs
  4. Severe, life-threatening (AIS = 4): moderate SDH, Grade IV liver lacerations.
  5. Critical (AIS = 5): total transection of the cervical spine, brainstem compression
  6. Non-survivable (AIS = 6): decapitation; complete transection of the aorta.

### Injury Severity – Physiological characteristics

This is Step One in the Field Triage Decision Scheme promoted by the American College of Surgeons (see Resources for the Optimal Care of the Injured Patient, 2006; CDC guidelines for the field triage of patients, 2009).

1. Glasgow Coma Scale. The CDC guidelines and rationale for field triage to a trauma center if the GCS is <13 is based on the idea that GCS is reasonably predictive of an ISS>15 (OR 7.7), the need for immediate surgical interventions, and the risk of death (case fatality rate of 24.7% with an GCS<14). ACS-COT further recommends the interfacility transfer of patients with a GCS<15 or neurological abnormalities, but with the modifier that the transfer is necessary only if the hospital does not have the resources necessary to manage the injury (see ATLS Student Course Manual 2004). In the game, I therefore focused on helping physicians to identify the subset of patients with severe TBI (i.e., patients whose brain injury would have resulted in intubation).
   1. It may be useful/necessary to review the components and calculation of the GCS with physicians at some point during the sessions.
   2. We should probably also review the categorization of TBI: 3-8 = severe; 9-11 = moderate; 13-15 = minor.
   3. The Brain Trauma Foundation recommends repeated use of the GCS in the pre-hospital and initial phase of treatment to screen for severe TBI (weak recommendation).
2. Shock: evidence of inadequate tissue perfusion and oxygenation. The CDC recommends the field triage of patients with a systolic BP<90 because of evidence that this level is associated with increased odds of major surgery/death (OR 14). The case fatality rate for these patients is 32.9%. The game recommends that the player triage patients using a similar threshold. The things we want to reinforce with trainees are:
   1. The reason for this dictate is that a single episode of hypotension during the prehospital or early hospital phases is associated with dramatic increases in mortality. **Patients with evidence of shock have an early mortality rate of 12% and a late mortality rate of 32% (see Zenati et al, 2002).** Hypotension becomes clinically apparent during Class III hemorrhage, when about 30% of the circulating blood volume is lost.
   2. There is no single test that diagnoses shock.
   3. The definition for 'hypotension' is controversial. The value of 90 mmHg has been used commonly as a threshold, particularly when considering 'permissive hypotension' until definitive hemorrhage control can be achieved. But analysis of outcomes in traumatic brain injury suggests that the association between systolic blood pressure and the adjusted probability of death is monotonic between 40-119 mm Hg, with each 10-point increase in systolic pressure associated with a decrease of 18.8% in the adjusted odds of death. (Spaite et al., 2017).
   4. Therefore, when in doubt, use the threshold of 110 to identify the widest cohort of patients.
3. Respiratory Failure. The field triage guidelines recommend the immediate transfer of patients with a RR<10 or ≥29. This feels a little nuanced to me, additionally the data is potentially a little more suspect (most studies do not disentangle the effect of hypotension and respiratory rate on outcomes), so instead in the game, this category is collapsed into patients who require intubation.

###

### Injury Severity – Anatomical characteristics

This is Step Two of the Field Triage Decision Scheme.

- The American College of Surgeons recommends the field triage of patients with: a) penetrating injuries to the head, neck, torso, extremities proximal to elbow/knee; b) flail chest; c) 2+ long bone fractures; d) crush, degloved, or mangled extremities; e) amputation proximal to wrist/ankle; f) pelvic fractures; g) open/depressed skull fractures; h) paralysis. ATLS has an even longer list of criteria for the interhospital transfer of patients (e.g., patients who may need prolonged mechanical ventilation, signs of great vessel injury etc) – which will be hard to make memorable.
- The game focuses on four groups of patients: a) penetrating injury to the torso; b) evidence of spinal cord injury; c) a mangled extremity; d) two or more body regions with a 'significant' injury.
  1. Penetrating injury to the torso. The CDC makes this recommendation because of the high likelihood that the patient will need rapid surgical intervention (they specifically reference the need for an emergency thoracotomy). They also state that the potential is high for severe injury and adverse outcomes because of vascular damage that might result in life-threatening exsanguination, and nerve damage that might result in permanent disability. Because management of these injuries might require skills and resources not available at every hospital, triage improves the likelihood of prompt access to trauma surgeons, cardiothoracic surgeons, vascular surgeons, orthopedic surgeons, and properly equipped ICUs and operating theaters.
  2. Spinal injury and amputation have a PPV of 100% for an ISS>15. The case fatality rates with paralysis and amputation are 7.1% and 10.1% respectively. In contrast, proximal long-bone fractures have a PPV of 19.5%, although the case fatality rate is quite high (8.8%). Rapid intervention is therefore required to prevent morbidity and mortality. I classified amputation more broadly into the category of 'mangled extremity' despite limited evidence. However, it is consistent with the CDC's consensus that injuries that deglove, crush or mangle extremities are complex and may threaten loss of limb. Treatment of vascular injury within six hours is the major determinant of limb salvage. Further, the risk for ischemia, wound infection, delayed or non-union of fractures, and chronic pain is high. Therefore, transfer to a trauma center provides the best change for appropriate and rapid assessment and treatment.
  3. Two or more body regions with a severe, non-life-threatening (AIS = 3) injury. This is the only criterion not explicitly derived from either the field triage guidelines or inter-facility triage guidelines. It came out of short interviews I conducted with a sample of national experts (n=6). It is based on the idea that this simple rule captures many patients with an ISS >15 or those with a 'severe injury' (see Copes et al, 1988). **It is further supported by evidence that patients with two injuries in different body regions have worse mortality (OR 1.75) than patients with two injuries in the same body region (see Moore et al, 2006).**

*Of note, the game does not include any effort to communicate data from Step 3 of the Field Triage Decision Scheme (screening patients based on mechanism of injury - falls, high risk auto crashes, auto v. pedestrian, motorcycle crashes). If it comes up, the PPV is very questionable for these criteria. Use of these screening criteria results in patients being mis-triaged between 39-84% of the time.

### Patient reserve

This is Step 4 of the Field Triage Decision Scheme.

1. Age.
   1. Age is a major predictor of under-triage (OR 0.48 [Chang et al., 2008]; OR 0.35 [Hsia et al., 2011]).
   2. Age is also a major predictor of mortality, with patients over the age of 75 being twice as likely to die as those from 65 to 74 (Hashmi et al., 2014).
   3. Older adults are increased risk of adverse outcomes after injury because of limited cardiovascular reserve, comorbidities, and general frailty (Bonne et al, 2013). Greater than 50% of the geriatric trauma population has underlying hypertension, and greater than 30% has heart disease. The comorbid conditions that confer the highest risk of mortality in the geriatric population are hepatic disease, renal insufficiency, and cancer. The presence of CHF can confer a 5- to 10-fold increased risk of death following trauma.
   4. **Elderly patients (even those with minimal injury) treated at high-volume trauma centers are less likely to experience preventable adverse events (Matsushima, 2014), and risk-adjusted mortality (Goodmanson, 2012). Early aggressive care with liberal use of ICU care, among patients older than 70, reduces mortality by 30% (Demetriades et al, 2002)**
   5. Presence of advanced age (age ≥65 or possibly age≥70) and pre-existing medical conditions should therefore lower the threshold for triage to a trauma center [Level II EAST guideline]. Presence of advanced age (age ≥65) and even one serious injury (AIS 3) should prompt treatment in the ICU of a trauma center [Level III EAST guideline].
2. Frailty.
   1. Defined as a syndrome of decreased physiological reserve and resistance to stressors. In trauma patients, it is associated with in-hospital complications (e.g., UTI or pneumonia), adverse discharge disposition (e.g., SNF), and in-hospital mortality, and is independent of age (Joseph, 2014) and co-morbidities (Fried et al., 2001).
   2. **There are multiple measures of frailty in trauma (MacDonald, 2016). However, the Fried frailty phenotype – slowed walking speed, low physical activity, unintentional weight loss, low energy, and low grip strength – is one feasible means of quickly, reliably, and validly assessing frailty in the ED or in the clinic. Presence of 3/5 criteria indicates frailty (Fried et al, 2001; Lee et al., 2017).**

### Hospital resources.

This is the concept that will require the greatest unpacking, and reinforcement. The interfacility triage guidelines state that physicians should "transfer when a patient's needs exceed available resources and are outlined in Table 13-1." However, the guidelines also include a caveat: "It is important to note that these criteria are flexible and must take into account local circumstances." As a result, there is likely a lot of ambiguity about which patients should be transferred and which ones can remain. In some studies, the characteristics of the non-trauma center (bed size, distance to a trauma center, proportion of Medicaid payer mix) are the strongest predictor for whether an injured patient is transferred to a trauma center from the ED (see Newgard et al, 2006, Mohan et al, 2015, Zhou et al, 2017), although a more recent paper suggests that accuracy is variable even after adjusting for hospital characteristics (Tillman et al., 2020). One of the things we need to help physicians do is to recognize whether their hospital has the resources required to treat patients.

- Gomez D et al., 2012 classified two different kinds of resources that exist at non-trauma centers: personnel (prevalence of EM-trained ED physicians or non-EM trained ED physicians; the availability of surgical resources [general surgery, orthopedic surgery, neurosurgery); physical resources (CT scanner, ICU). They classified hospitals as resource rich (all human/physical resources were available), resource intermediate (some but not all resources available), resource poor (no resources available). **The authors also went on to assess outcomes of patients taken initally to NTCs (Haas B et al, 2012). The set of papers found that resource rich hospitals are half as likely to transfer patients (transfer drops from 50% to 27%), and this translates into worse outcomes (OR of 48 hour mortality is 0.68).**
- The game's objective is to teach physicians that when patients are on the bubble (i.e., are young but have moderate injuries), that transfer should depend on whether the hospital can adequately address their injuries.

## Pedagogical Strategies

We list below in Table 3 the pedagogical strategies that we think coaches may find useful as they progress through the coaching sessions. The strategies are drawn from Doug Lemov's book *Teach Like a Champion 3.*0. The table includes definitions, as well as examples of how to use these concepts during specific situations. We provide additional specific examples of how to use these strategies in the section titled "Structuring the Three Sessions."

**Table 3.** List of pedagogical strategies

| **Pedagogical strategy** | **Description of strategy** | **Examples and/or sample prompts** |
| --- | --- | --- |
| Planning for error | A strategy of anticipating predictable mistakes made by trainees, and preparing a response in advance. Preparation not only increases the rate of recognition of these errors but also increases the likelihood of a productive response. | **Level 2** - Multi-system trauma. Trainees frequently struggle with the question of "similarities" between the cases because of its' abstractness. The coach should have a strategy in mind to handle these situations. For example, the coach can use a *break it down* approach: "what is one reason you might transfer Ms. Douglas...okay what is another reason...?" |
| Questioning | A strategy for phrasing questions so that they begin with perception and then move to knowledge building and finally to checking for understanding. | Perception - "what do you see?"  Knowledge building - "what does it mean?"  Understanding - "what is our objective here?" |
| Exemplar planning | A lesson plan with correct answers to the questions that the coach will ask. If agreement on the response is established before the start of the coaching session, it reduces variability in execution and preserves autonomy. | See section "Modelling Document." Ideally the coach will addend the information based on their own experience with the game. |
| Creating a culture of error | A strategy for encouraging trainees to think of the 'wrong' answer as the first, positive, and critical step toward getting it 'right, socializing them to acknowledge and to share mistakes with interest and fascination. | "Remember that we want you to make mistakes...mistakes allow us to identify opportunities to make sure that we are being clear." |
| Active observation | A strategy of prioritizing the recognition of specific errors that commonly compromise performance, by actively tracking their occurrence. This strategy improves analysis of errors of judgment and can improve the quality of the feedback provided to the trainee. | **Level 3** - triage of young patients with moderate injuries depends on the resources available at the non-trauma center (severity of injury + age + resources). A typical error is to mis-categorize the severity of the injury as either minor (≥2 rib fractures) or severe (femur fracture), and from there fail to pay attention to age and/or hospital resources by recommending discharge or transfer. |

# Curriculum

## Overview of structure of coaching sessions

The coach will meet the trainee for three thirty minute sessions (one per week) using a video conferencing platform (e.g., Zoom). During these sessions, the trainee will play 1-2 levels of *Shift with Friends*, and the dyad will discuss trauma triage decision making.

|  | **Session 1** | **Session 2** | **Session 3** |
| --- | --- | --- | --- |
| **Rule of play** | Improve their pattern recognition | | |
| **Sub-rule of play** | Identify several classes of severe injuries:   - Patients who have a penetrating injury to their torso have a severe injury - Patients in shock have a severe injury | Identify the importance of patient reserve in triage decisions:   - Patients with limited reserve (i.e., older adults) will need resources only available at quarternary care centers.   Identify 1 more class of severe injuries*:   - Patients with two or more body regions have severe injuries.   **conditional on what was covered in Session 1* | Identify the importance of patient reserve in triage decisions:   - Patients with limited reserve (i.e., frail adults) will need resources only available at quarternary care centers.   Identify the importance of hospital resources in triage decisions:   - Young patients with serious injuries may need resources available at quarternary care centers. - Patients with minor injuries rarely need the resources available at trauma centers. |
| **Game levels to be covered**  *Letter denotes difficulty of the level: E=easy; M=moderate; H=hard | Level 8: shock (E)*  Level 1: penetrating (E) - covered by coach | Level 2: 2+ body systems (M)  Level 9: old + serious (M)  OR  Level 7: mangled extremity (E)  Level 2: 2+ body systems (M) | Level 9: old + serious (M)  Level 10: frail + serious (M)  OR  Level 10: frail + serious (M)  Level 3: young + serious + limited hospital resources (H) |
| **Skill acquisition** | 1. Pattern recognition – differentiating between patients who must GO and those who should STAY. 2. Cognitive barriers – why should this case GO vs. STAY 3. Institutional barriers – what would keep you from acting on your judgment that this case should GO vs. STAY. | | |
| **Technical vocabulary** | Why should we transfer trauma patients?  Patient recognition – physiologic characteristics | Severity of injury classification  Patient reserve | Patient reserve  Hospital resources |
| **Learning objectives** [with Bloom's taxonomy in parenthesis] | 1. Categorize injury severity as minor, moderate, serious, severe using AIS [*Analyze]* 2. Justify transfer decisions using three contextual cues: **injury** **severity**, patient reserve, hospital resources. [*Evaluate*] 3. Create two triage decision principles: patients with shock and penetrating injuries to the torso should be transferred to a trauma center [*Create*] | 1. Justify transfer decisions using three contextual cues: injury severity, **patient reserve**, hospital resources. [*Evaluate*] 2. Use markers of patient reserve (age/frailty) to make triage decisions. [*Apply*] 3. Create two decision principles: patients ≥70 or should be transferred to a trauma center; patients with mangled extremities and ≥2 systems should be transferred to a trauma center. [*Create*] | 1. Justify transfer decisions using three contextual cues: injury severity, patient reserve, **hospital resources**. [*Evaluate*] 2. Compare patients where hospital resources should or should not influence triage decisions [*Analyze*] 3. Create two decision principles: young/serious/minimal resources - transfer; frail/serious ­– transfer. [*Create*] |

## Structuring the Three Sessions

### Notes to the coaches

- There are four things that coaches need to accomplish:

1. Share responsibility and contribute to equal exchange
2. Use questions/prompts to guide trainee's self-reflection and analysis
3. Provide constructive feedback and encouragement
4. Guide goal setting and action planning.

- It is okay to deviate from the curriculum, conditional on the expertise of the trainee. If they are very experienced, it is fine to skip to the more challenging levels (e.g., Level 3). If they are struggling, it is fine to return to the easier levels (e.g., Level 1).
- There will be different types of learners. The goal is to enlist them all as our partners in this enterprise. We want to create a culture of error, where mistakes represent an opportunity to make teaching points.
  - Compliment them on their questions and try to be as positive as possible throughout the encounter.
  - De-personalize negative actions or performance during the game.
- Customize the sessions based on the learners' context and goals
- Discuss real-world applications and challenges when possible.
- Useful pedagogical strategies are highlighted in bold. There are also sample scripts and prompts provided below.
- Remember coaches should attempt to perform active observation and should plan for error.
- Questioning should move from perception to knowledge to understanding.

### Session 1

- Introduction - develop the relationship. Set out your credentials.
- Get a sense of their current environment and opinions about trauma triage - where are they practicing; what resources do they have; who admits their patients currently. Try to do three things:
  - *Guide their goal setting*:
    - Sample script: What are some reasons that you think that people would participate in this kind of exercise? What could we provide that would make participation worthwhile?
  - *Enlist them as partners in the endeavor*:
    - Sample script: This is our unrefined first draft. At the end we are going to stop and get your feedback on how to improve our training manual. It is so nice of you to volunteer to do this. It's great that you are willing to help us learn how to run a virtual coaching intervention.
  - *Understand their prior experience transferring patients*:
    - Sample script: Tell me a little about the hospitals at which you work? Tell me about your systems of care? Do you routinely transfer trauma patients? How does the transfer process usually go? Again, link what they say to their goals OR to the reasons why they think others might be interested in a coaching intervention.
- Describe the purpose of the game and the background of the project
  - Disseminating clinical practice guidelines in trauma triage.
    - Sample script: We are attempting to modify pattern recognition so that it aligns better with the guidelines – not trying to eradicate pattern recognition but just broaden the things that people consider as indicating someone who may benefit from transfer to a trauma center.
  - Link back to the participant's stated goals
    - Sample script: We are trying to simplify the decision process
    - Sample script: We are trying to provide some of the technical language you can use when you are communicating with trauma centers.
- Introduction of game mechanics and environment of the game.
  - Have they set up their iPad and downloaded *Shift with Friends*? If not, then pause for them to do this.
  - Have they created their login and password? If not, pause for them to do this. The website is: https://decisionmaking.ccm.pitt.edu. They will need to create an 8 digit alphanumeric password with at least one capitalized letter and one special character. Once they have been granted access, then they will get an email. [**WORST CASE SCENARIO**: you can share your login/password].
  - Review the game mechanics. Share your screen (instructions in the introductory document "What to Expect") and start with Hospital 1 (penetrating injury).
    - Things to highlight before you start: 1) each level occurs at a different type of hospital. Information about hospital characteristics is provided on the introduction page – the only level where they will see more than one hospital is Level 3 – and is also present under case information; 2) we want them to "think aloud." Please model this behavior for them. Let them know that if they find it distracting to speak as they think, they can summarize after they make their decision.
    - Things to highlight once the level starts: 1) the clock - 90 seconds to review 10 cases. Set expectations early ("most people complete between 2-4 cases the first time they play a level...this is normal...don't worry about the snarky feedback within the game"); 2) the magnifying glass - need to click once to explode a clue and to click again to close it, otherwise it will obscure other information; 3) make decisions based on only as much information as you need.
    - Emphasize that the goal is to focus on pattern recognition.
- Start reviewing Level 8 (shock). Each round should take about 5-10 minutes
  - Triage cases (allow them to do this independently) – 90 seconds.
    - Preface this by emphasizing that the focus is not on getting the cases 'right.' For example, "We want you to make mistakes. Otherwise, you will put me out of a job. Help me figure out where our colleagues might have opportunities for improvement."
    - Remember to try to perform active observation – you are looking for predictable errors that they might be making (see "Modelling Document - Details of the Level").
    - After the first set of cases. Ask them to explain their decision making. Be as non-judgmental as possible. Don't provide them with any clues about whether you think they are right or wrong. "Tell me more about that?" "Okay, help me to understand that."
    - Then ask them to triage the second set of cases (if they haven't gotten through >5 cases the first time). Provide a focus for their attention. Reinforce that this is about pattern recognition.
    - When their thought processes are unclear during think-aloud, probe with specific questions afterwards. "You mentioned blood pressure a few times during that round of triage. Can you tell me more about how that information influenced your judgment?"
  - Second session of triage. Almost no one is getting through 4 cases in their first set of triage, so prior to reviewing more cases, potentially pause and provide a focal point in advance.
    - Sample script: "we are going to run through a second set of cases. Remember to pay attention to the physiologic status of the patient."
  - Initial feedback
    - Some people become upset by the cases that they get wrong. If it seems indicated, you can pause for a minute to discuss why that is the case.
  - Structured case comparison. Pause again when player gets to this step.
    - Asking for similarity seems to be challenging. May need to explain the construct further: "we are looking for commonalities among patients (big, broad commonalities) that might inform a rule about triage."
    - Remind them that each level focuses on one rule - one thing that they should remember.
    - Pose perceptual question - what do you think is most important about this case? what makes you think that?
      - Recognizing that there are multiple cues that might seem salient, we want to respond to their answers with encouragement. Emphasize what they got right and prompt them to consider additional options.
        - Great. What are other reasons for transfer?
        - Great. What might have caused that.
        - Here is some information about what the experts think.
    - Follow by knowledge building questions. For example, "what does this mean about our triage principle?"
    - Conclude with an understanding question. For example, "what might happen if you were not able to transfer this patient?" or "what might keep you from transferring this patient?" [Potentially provide personal anecdotes about patient outcomes...positive/negative]
    - Consider providing an exemplar. "What you see here is that this patient is hypotensive, so regardless of what other information you get, he needs to be transferred."
    - Provide rationale and discuss *technical vocabulary*. For example, "I want to pause for a second to talk about a couple of key ideas." Concepts to review here are:
      - [Patient recognition](#_Patient_recognition_1)
      - [Physiological characteristics #2 – shock](#_Physiological_characteristics)
  - Summative feedback. Discuss the insights from the level(s) covered. Try to use language that emphasizes the relatedness of the ideas being discussed (habits of discussion).
    - "I want to build on what you said."
    - "I understand why you would say that, but..."
  - Ask the trainee to reflect on one thing surprised them. And one thing that they think they should practice during the next session or that they want to think about.
  - Provide reminder about next session. Emphasize importance of being present (try to lean into altruism and relatedness as core values of this project).

### Session 2

- - Move to a new level (pick based on what they remember): if they do not remember, then return to Level 8. If they do remember, then ask them if they are interested in starting with something abstract or something more concrete.
    - If they want to try something concrete: start with Level 7
    - If they want something more abstract: start with Level 2.
  - Move to new level:
    - Level 8 --> Level 2 or Level 7
    - Level 2 --> Level 9
    - Level 7 --> Level 2
  - Put the session into context.
    - If starting with Level 2. We are trying to build pattern recognition and that means helping you to categorize injury severity when you meet a patient. We have spoken about some mechanisms/physiologic parameters that you can use as a shortcut to identify severe injuries. Today we will be covering another shortcut.
    - If starting with Level 8 or Level 7. Same language...but in this case emphasize the fact that we are using physiologic parameters as short cuts.
  - Technical vocabulary to be covered in this session:
    - [Injury Severity – Anatomical characteristics](#_Injury_Severity_–) (after Level 2 or Level 7)
    - [Patient reserve.](#_Patient_reserve.) (after Level 9)
  - Continue building rapport based on past exchanges/knowledge of the participants.
  - Provide reminder about the next session.

### Session 3

- - Plan to cover 2 levels.
    - Level 10 and Level 3
    - Level 9 and Level 10
  - Summarize the things that we covered.
    - Reinforce that we are interested in pattern recognition. The levels of the game are designed to help the player to break injuries into three categories: minor (never have to go), moderate, and severe (always goes). And we have covered some of the things that modify when moderate injuries have to go to a trauma center (age, frailty, *resources that are available).
    - Tailor this conversation to the stuff that was covered.
  - Technical vocabulary to be covered in this session
    - [Patient reserve](#_Patient_reserve) (after Level 10 or Level 9)
    - [Hospital resources](#_Hospital_resources.) (after Level 3)
  - Post-coaching session tasks:
    - Ask them to complete the assessment of the coaches' performance online.
    - Provide reminder about virtual simulation.
    - Let them know that we will be scheduling debriefing interviews.

# Modelling document

## Overview

- There are ten primary levels - each representing a different decision principle. We can categorize the levels: 6 are easy, 3 are moderate difficulty, and 1 is hard.

| **Level** | **Principle** | **Difficulty** |
| --- | --- | --- |
| 1 | Penetrating | Easy |
| 2 | Multiple systems | Moderate |
| 3* only level that highlights dissimilarities | Moderate injuries + hospital resources | Hard |
| 4 | Intubation | Easy |
| 5 * only level that emphasizes not to transfer some patients | Minor injuries | Easy |
| 6 | Paralysis | Easy |
| 7 | Mangled extremity | Easy |
| 8 | Shock | Easy |
| 9 | Elderly patients + moderate injuries | Moderate |
| 10 | Frail patients + moderate injury | Moderate |

- The progression of the game loop is: 1) triage 10 cases in 90 seconds (5 transfer/5 do not transfer); 2) compare two of the 5 cases that belong in one set); 3) triage 10 cases in 90 seconds (optional); 4) generate decision principle for the level. Figure 1 shows how this works in practice.

| **Figure 2. Screenshots of game loop** | | |  |
| --- | --- | --- | --- |
| **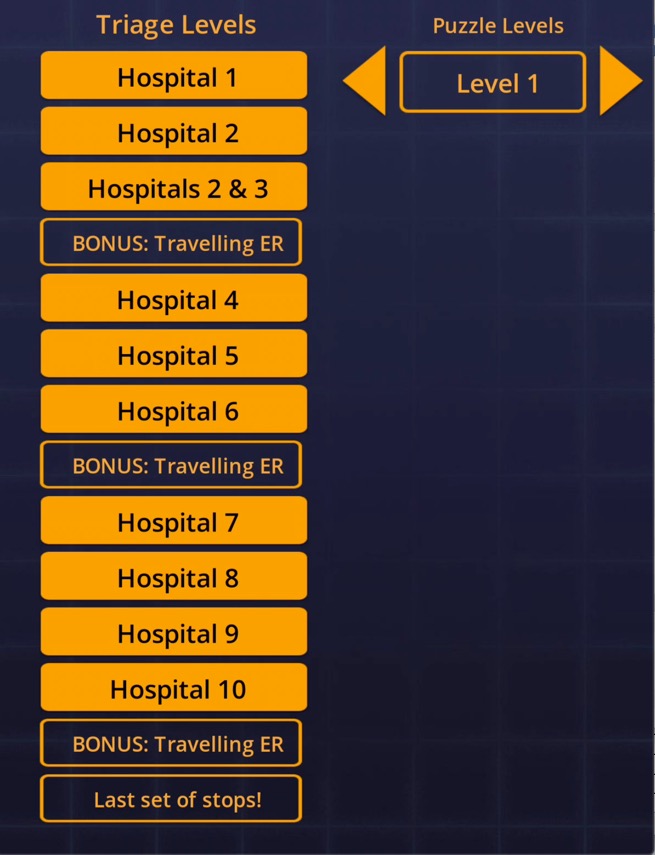** | **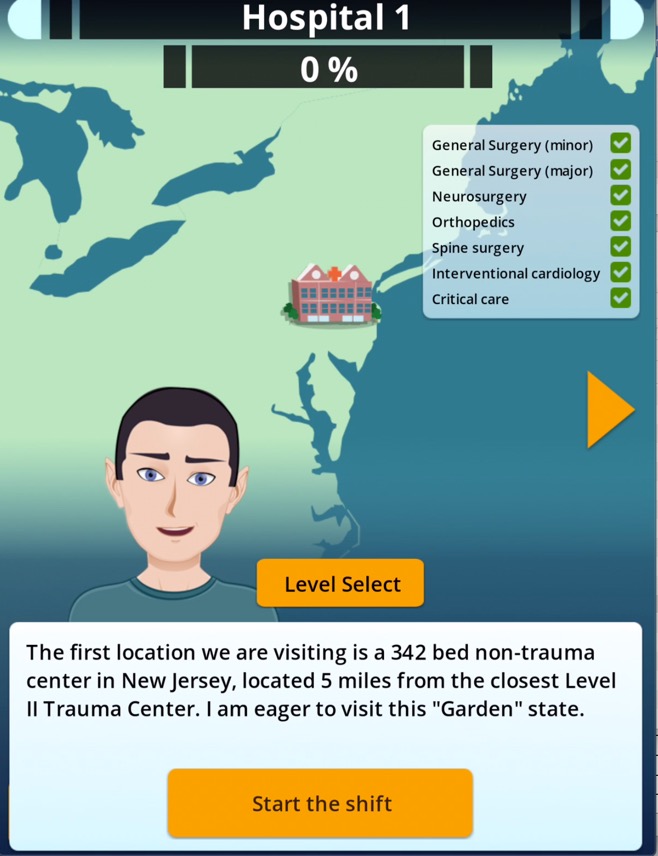** | **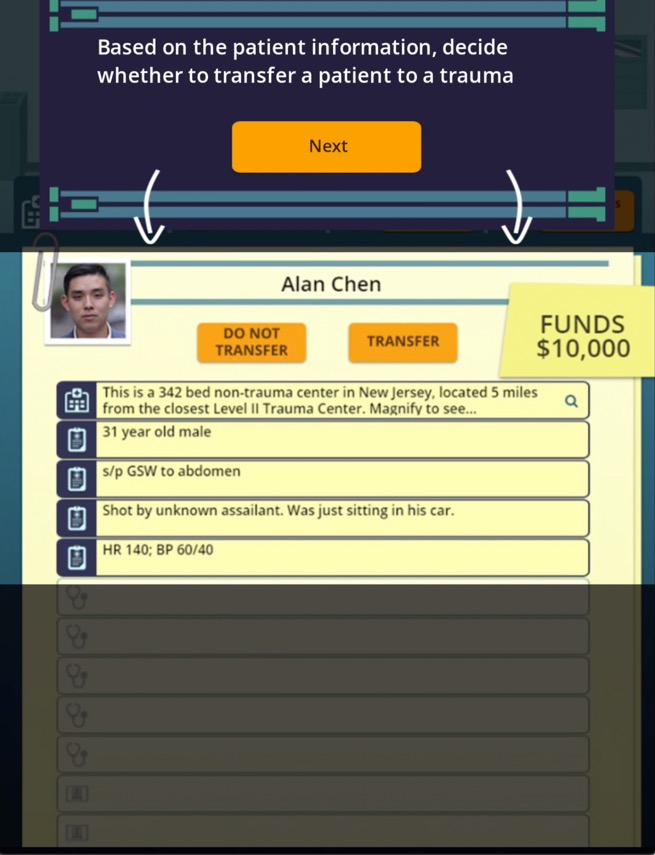** | **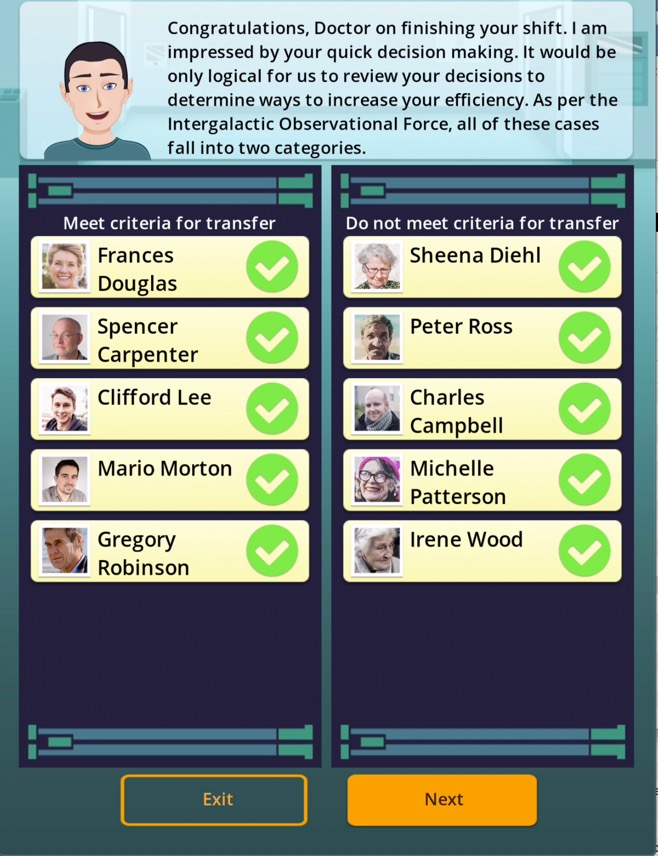** |
| **Level Select** | **Level Home** | **Case Triage** | **Feedback - triage** |
| **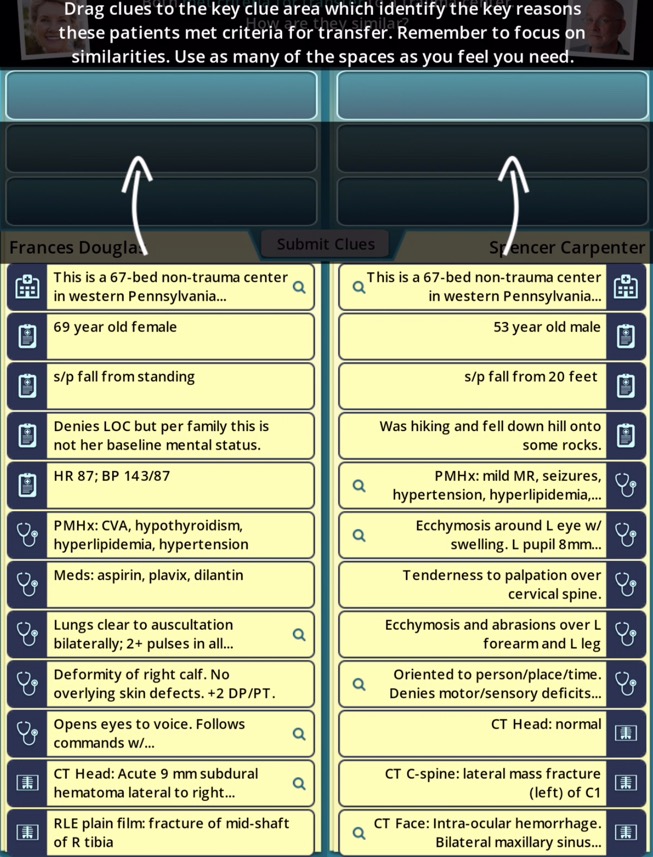** | **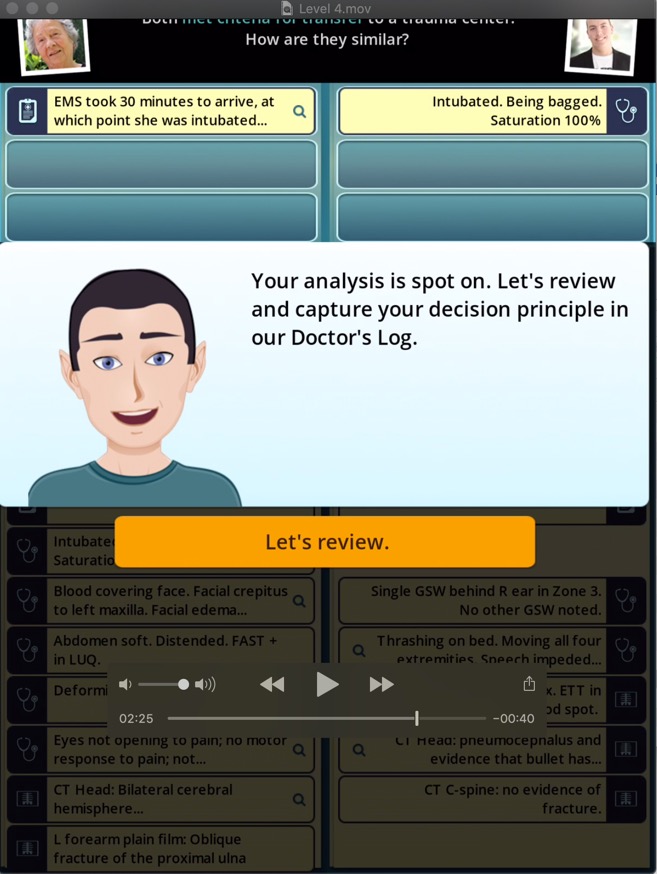** | **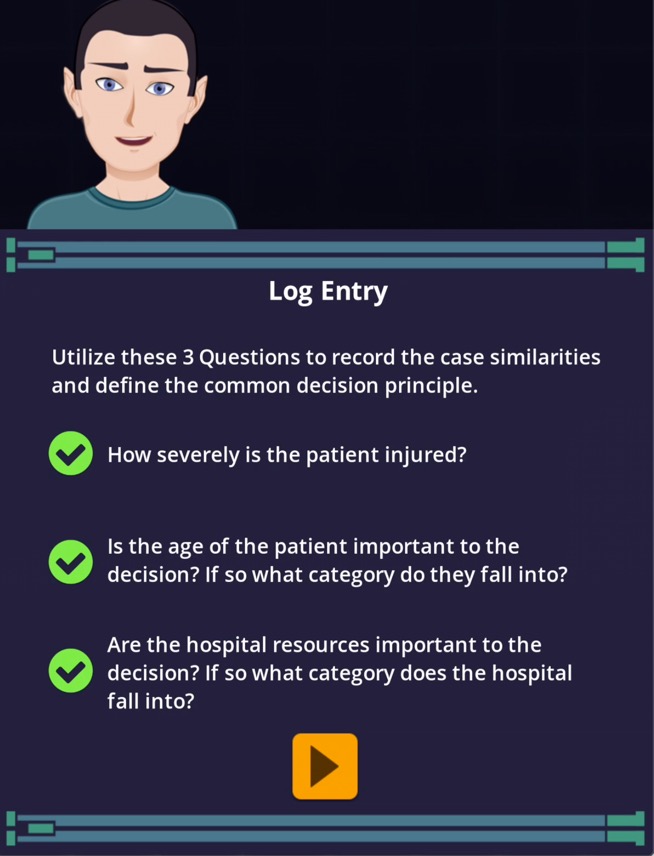** |  |
| **Case comparison** | **Feedback - comparison** | **Generate decision principle** |  |

- If you get all the cases correct, and identify the right cues for the comparison step, the option of a second round of triage is not offered.
- You must get a certain number of cases right (>6) during triage to progress to the comparison step.
- There are three bonus rounds. These do not have a defined decision principle associated with them. Instead, they mix and match decision principles.
- You will see cases repeat across rounds - it's happening more frequently than we specified, and think it is a bug that they missed. It's not game blocking so don't think there is anything we can do about it now.
- Each level is located at a specific kind of hospital. Characteristics of the hospital are specified for each case (you need to click the information button). In addition, they are specified on the Level Home screen (i.e., the screen you see after you click the level you want to play).
- There is still the option for players to see the Match 3 puzzle (if that is of interest to them). The player just has to click the button under the Puzzle Level. The core mechanic of the puzzle game is adopted from popular casual games like *Candy Crush*, which is the connect the dots mechanic. There are set number of moves and points that you can get per move with a minimum/maximum per round. There are 10 levels, and as you move through the rounds the logic of the puzzle becomes more challenging.

| **Figure 3. Screenshots of optional game mechanics** | |
| --- | --- |
| **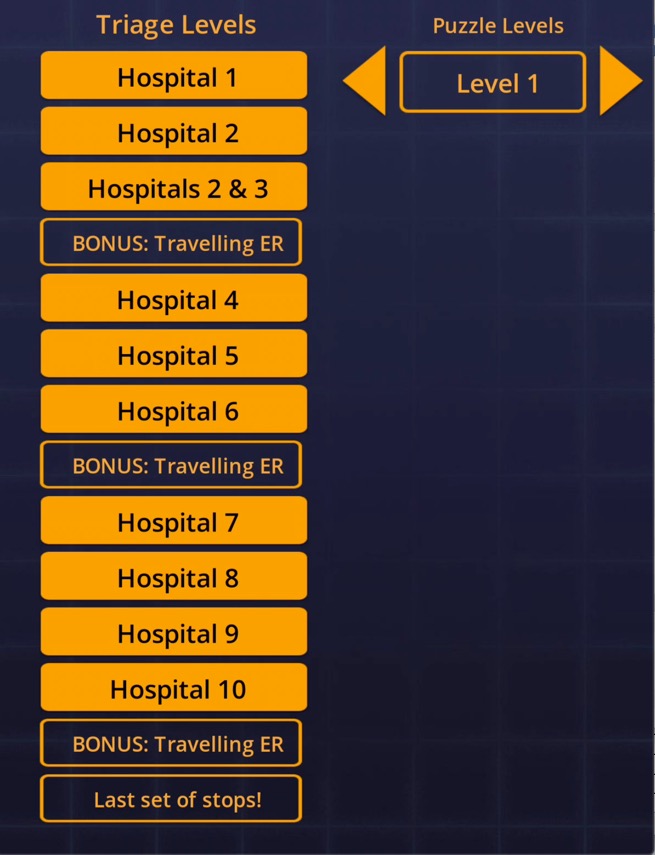** | **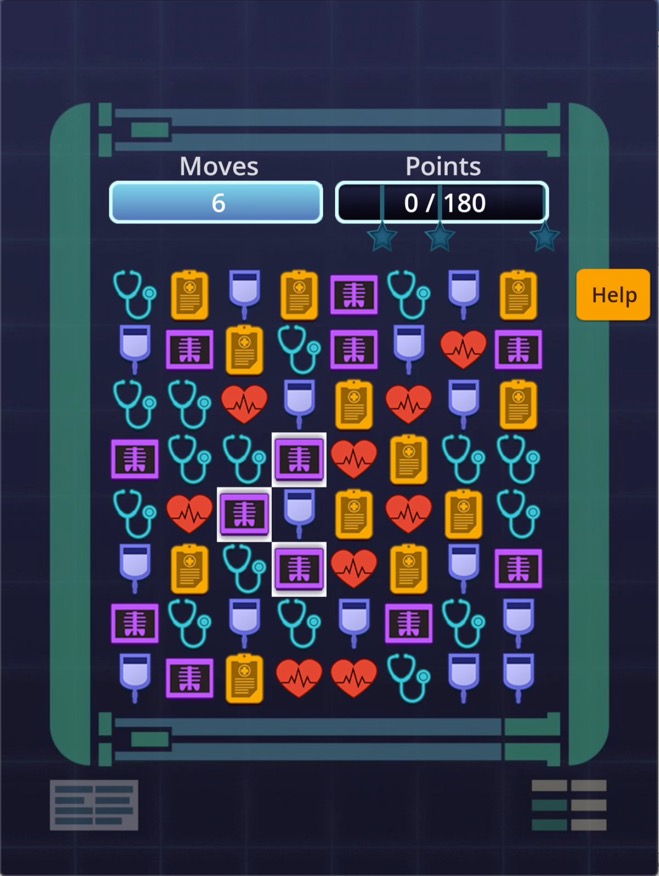** |
| **Level Select** | **Level 1 Puzzle Game** |

## Details of each level

### Level One

| Decision Principle | Transfer patients with penetrating injuries to the head, torso, or proximal extremities to trauma centers. |
| --- | --- |
| Difficulty | Easy |
| Bugs | None |
| Case comparison step - the answer is: s/p GSW to abdomen (Chen) and s/p GSW to abdomen and right arm (Marshall).  Prompt:   - what do you think is most important about this case?   Responses:   - Great. What are other reasons for transfer? - Great. What might have caused that? - Here is some information about what experts think.   Prompt:   - what do you think this means about our triage principle?   Prompt:   - what might keep you from transferring this patient? | 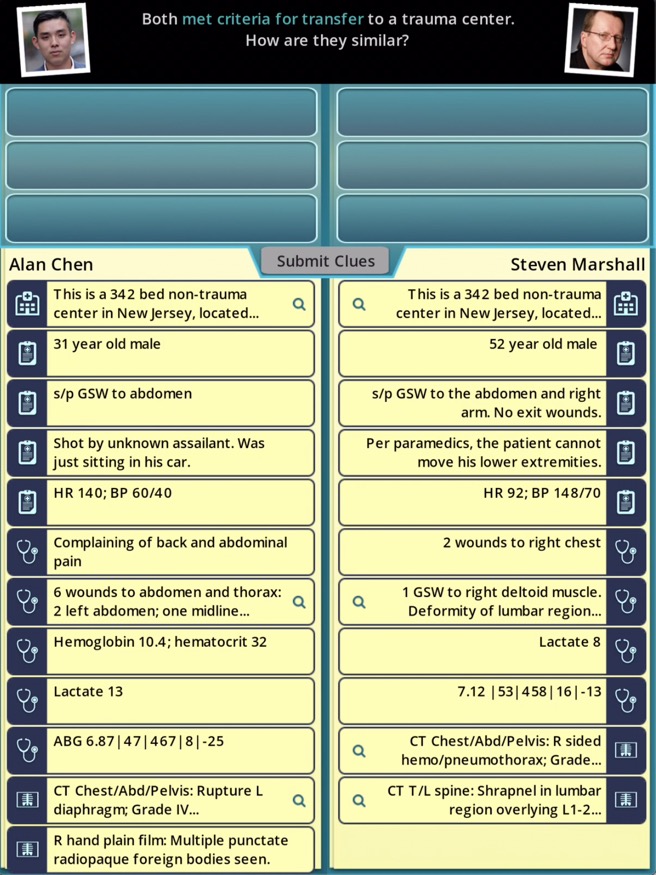 |
| What are predictable errors that might occur? | - Focus on hypotension for Chen   - That is absolutely correct and is a reason for transfer. What are some other reasons that the cases share? - Focus on the lactate/base deficit   - Great. What are some other reasons? - Focus on the CT scan findings.   - Gently remind them that patients who are shot, SHOULD NOT get scans at non-trauma centers – it extends their stay by an average of 73 minutes (Mohan et al., 2011). |
| Case review step | These patients have severe injuries. Age and hospital resources are immaterial |

| Level Two |  |
| --- | --- |
| Decision Principle | Transfer patients with injuries involving more than one body region to trauma centers |
| Difficulty | Moderate |
| Bugs | None |
| Case comparison step - the answer is: evidence of TBI and extremity injury - either radiographic or PE (Douglas) and evidence of cervical spine and facial fracture - either radiographic or PE (Carpenter).  Prompt:   - what do you think is most important about this case?   Responses:   - Great. What are other reasons for transfer? - Great. What might have caused that? - Here is some information about what experts think.   Prompt:   - what do you think this means about our triage principle?   Prompt:   - what might keep you from transferring this patient? | 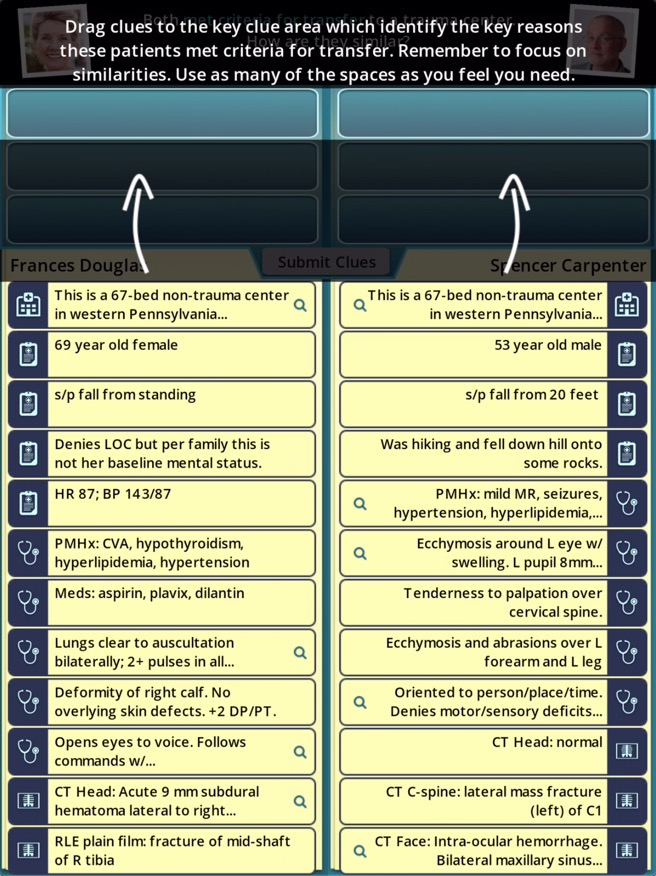 |
| What are predictable errors that might occur? | - Not recognizing that two moderate injuries combine to become one severe injury. - Not recognizing that injuries are moderate and not severe. - Not being able to grasp the abstract form of the question about similarities. |
| Potential prompts | - Back into the question of similarities by asking them to identify reasons for transfer for each case. - If they simply cannot get the right answer, then give them the rule, and then ask them to identify the injuries that make the rule true. |
| Case review step | These patients have severe injuries.  Age and hospital resources are immaterial. |

| Level Three | Listed as Level 2 and 3 (to indicate this is a step that highlights dissimilarities). |
| --- | --- |
| Decision Principle | The triage of young patients with moderate injuries should depend on the resources available at the non-trauma center |
| Difficulty | Hard |
| Bugs | When the level opens, you should see descriptions for two hospitals, but only one appears. However, if you click the advance/backward buttons, when you move back to the Level Home, both hospitals now appear. |
| Case comparison step - the answer is: rib fractures + size of the hospital (Cox) compared with femur fracture (PE or radiographic evidence) + large non-trauma center (Molinari).  Prompt:   - what do you think is most important about this case?   Responses:   - Great. What are other reasons for transfer? - Great. What might have caused that? - Here is some information about what experts think.   Prompt:   - what do you think this means about our triage principle?   Prompt:   - what might keep you from transferring this patient? | 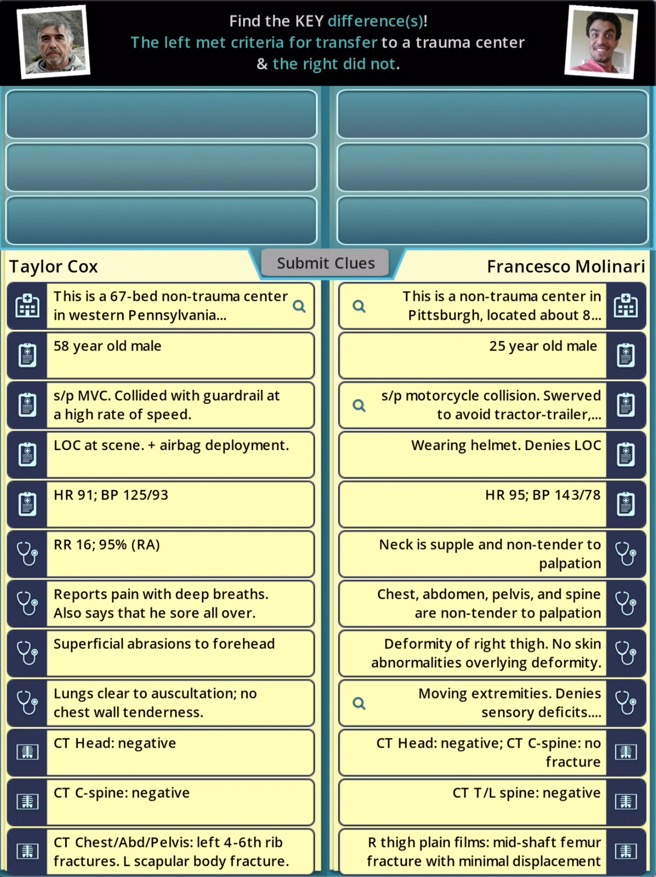 |
| What are predictable errors that might occur? | - Failure to recognize that ≥2 rib fractures and a femur fracture qualify as moderate injuries. Given the age of the patients (<70), the next important criterion has to do with the resources available at the hospital. |
| Case review step | Moderate injury + young patients + low vs. moderate hospital resources --> triage decision |

| Level Four |  |
| --- | --- |
| Decision Principle | Transfer intubated patients to trauma centers |
| Difficulty | Easy |
| Bugs | None |
| Case comparison step - the answer is: patients were intubated (clues #4 or #6 for both cases).  Prompt:   - what do you think is most important about this case?   Responses:   - Great. What are other reasons for transfer? - Great. What might have caused that? - Here is some information about what experts think.   Prompt:   - what do you think this means about our triage principle?   Prompt:   - what might keep you from transferring this patient? | 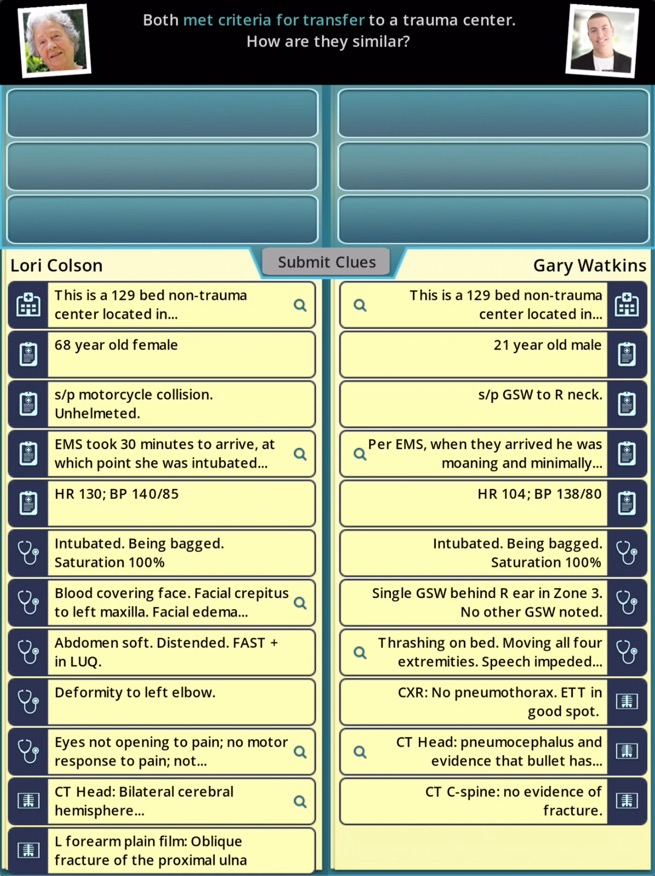 |
| What are predictable errors that might occur? | - Focus on severe traumatic brain injury as an alternative cue for transfer.   - This is correct. What are some other commonalities shared by the two cases? - Focus on penetrating injury for Watkins.   - This is correct and would indicate a reason for transfer for this patient. We are looking for reasons shared between the two cases to build a generalizable principle for transfer. |
| Case review step | Severe injury. Therefore age and hospital resources are immaterial. |

| Level Five |  |
| --- | --- |
| Decision Principle | Patients with minor injuries should not be transferred to trauma centers |
| Difficulty | Easy |
| Bugs | None |
| Case comparison step - the answer is: Diehl has a wrist sprain (tenderness on palpation and normal film); Ross has two lacerations to his foot (wounds to L foot, negative films). Therefore they both only have minor injuries.  Prompt:   - what do you think is most important about this case?   Responses:   - Great. What are other reasons for transfer? - Great. What might have caused that? - Here is some information about what experts think.   Prompt:   - what do you think this means about our triage principle?   Prompt:   - what might keep you from transferring this patient? | 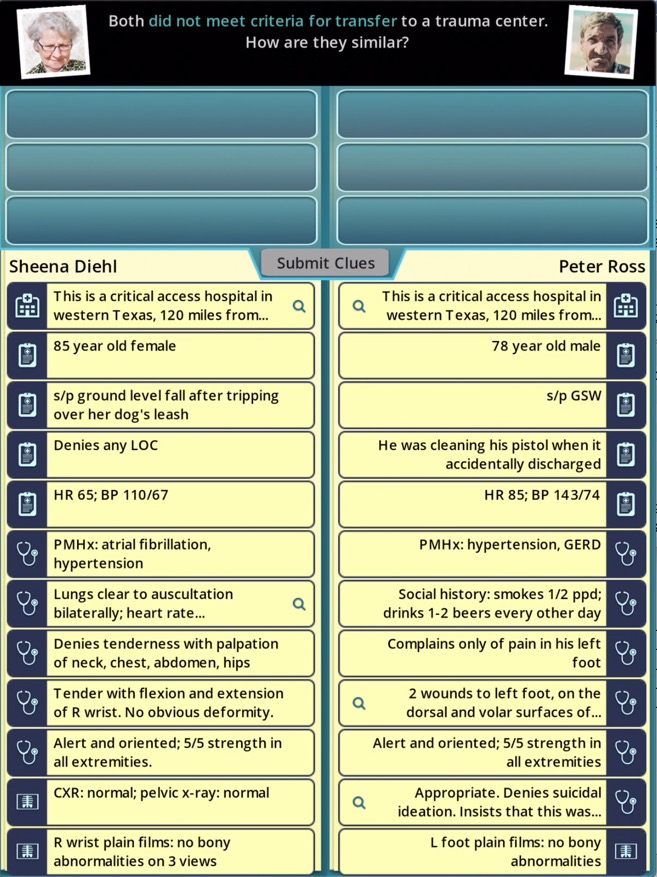 |
| What are predictable errors that might occur? | - Focus on the size of the hospital - critical access in Texas. - Gunshot wound in the case of Peter Ross. In this case it is below the level of the thigh so not severe. Also it is not a commonality shared between the cases. - Patients' ages: both are older than 70. |
| Case review step | Minor injuries. Therefore age and hospital resources are immaterial. |

| Level Six |  |
| --- | --- |
| Decision Principle | Transfer patients with paralysis or lateralizing neurological signs to trauma centers |
| Difficulty | Easy |
| Bugs | None |
| Case comparison step - the answer is: evidence of lateralizing neurological symptoms (Not moving lower extremities [Rogers] and loss of sensation below the waist [Stewart]  Prompt:   - what do you think is most important about this case?   Responses:   - Great. What are other reasons for transfer? - Great. What might have caused that? - Here is some information about what experts think.   Prompt:   - what do you think this means about our triage principle?   Prompt:   - what might keep you from transferring this patient? | 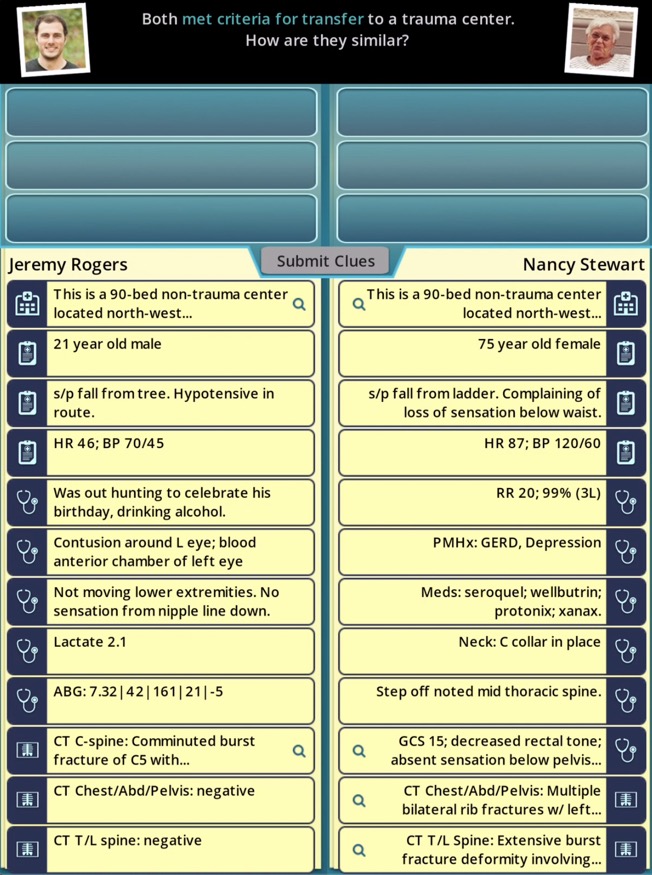 |
| What are predictable errors that might occur? | - Hypotension (Rogers) - More than one system affected (Stewart) - Age (Stewart) - Hospital resources - relatively small with no spine surgeon on staff. |
| Case review step | Paralysis or lateralizing neurological symptoms indicate a severe injury. Therefore age and hospital resources are immaterial. |

| Level Seven |  |
| --- | --- |
| Decision Principle | Transfer patients with mangled extremities to trauma centers |
| Difficulty | Easy |
| Bugs | None |
| Case comparison step - the answer is: evidence of mangled extremity (deformity of L upper arm or unable to wiggle fingers [Stewart] and deformity of tib-fib [Gray])  Prompt:   - what do you think is most important about this case?   Responses:   - Great. What are other reasons for transfer? - Great. What might have caused that? - Here is some information about what experts think.   Prompt:   - what do you think this means about our triage principle?   Prompt:   - what might keep you from transferring this patient? | 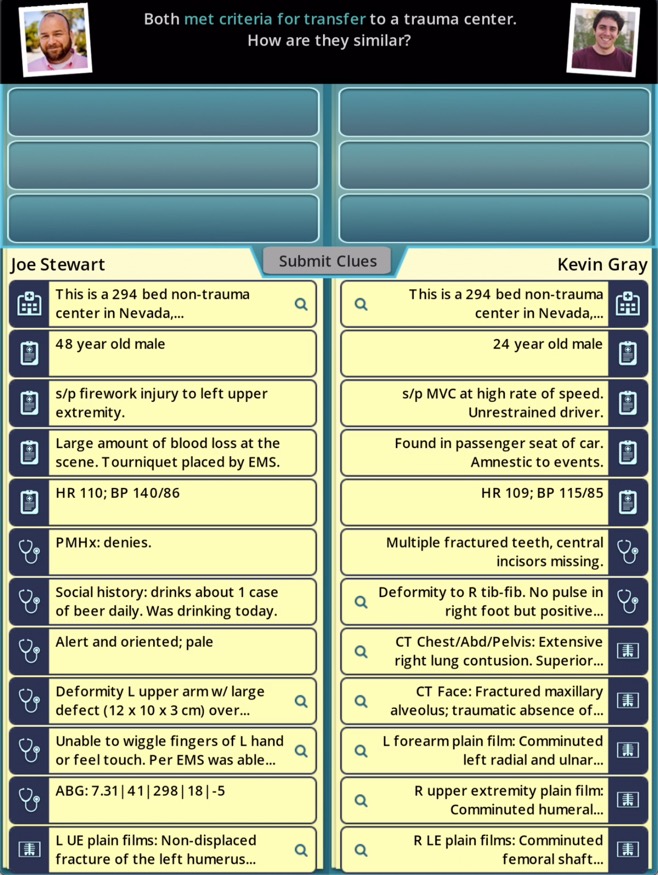 |
| What are predictable errors that might occur? | - Both look a little shocky - with tachycardia and in the case of Stewart an elevated base deficit. - Multiple systems affected (Gray) - Mechanisms for both are concerning: firework injury and MVC with high rate of speed. |
| Case review step | A mangled extremity denotes a severe injury. Therefore age and hospital resources are immaterial. |

| Level Eight |  |
| --- | --- |
| Decision Principle | Transfer patients in shock to trauma centers |
| Difficulty | Easy |
| Bugs | None |
| Case comparison step - the answer is: BP<90 for both cases.  Prompt:   - what do you think is most important about this case?   Responses:   - Great. What are other reasons for transfer? - Great. What might have caused that? - Here is some information about what experts think.   Prompt:   - what do you think this means about our triage principle?   Prompt:   - what might keep you from transferring this patient? | 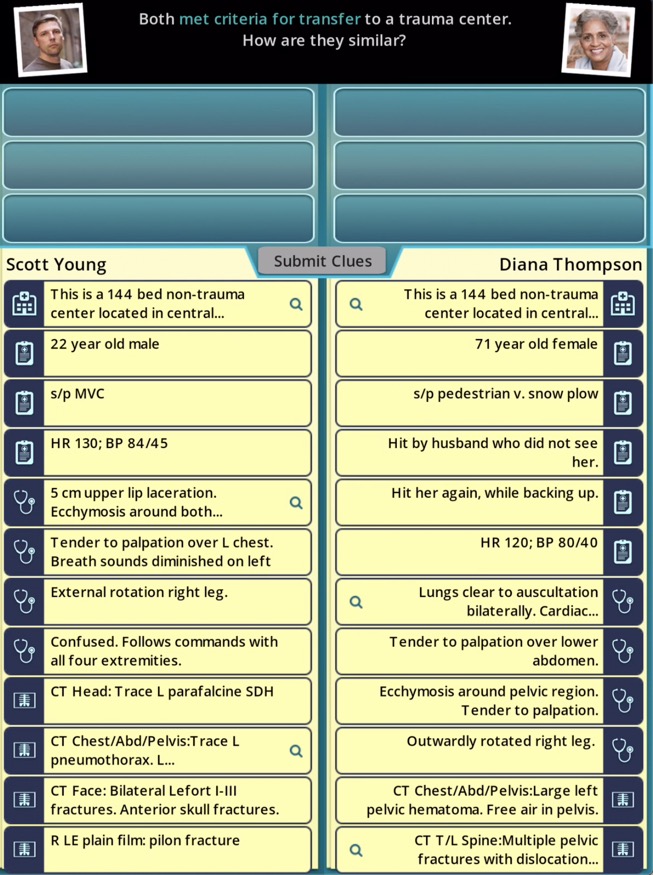 |
| What are predictable errors that might occur? | - Attention to the specific injuries in question and the fact that both patients have multiple systems affected. |
| Case review step | Shock denotes severe injury. Therefore age and hospital resources are immaterial. |

| Level Nine |  |
| --- | --- |
| Decision Principle |  |
| Difficulty | Transfer elderly patients (>70) with moderate injuries to trauma centers |
| Bugs | Moderate |
| Case comparison step - the answer is: age + evidence of moderate injuries (e.g., rib fractures [Phillips], TBI [Maryati])  Prompt:   - what do you think is most important about this case?   Responses:   - Great. What are other reasons for transfer? - Great. What might have caused that? - Here is some information about what experts think.   Prompt:   - what do you think this means about our triage principle?   Prompt:   - what might keep you from transferring this patient? | 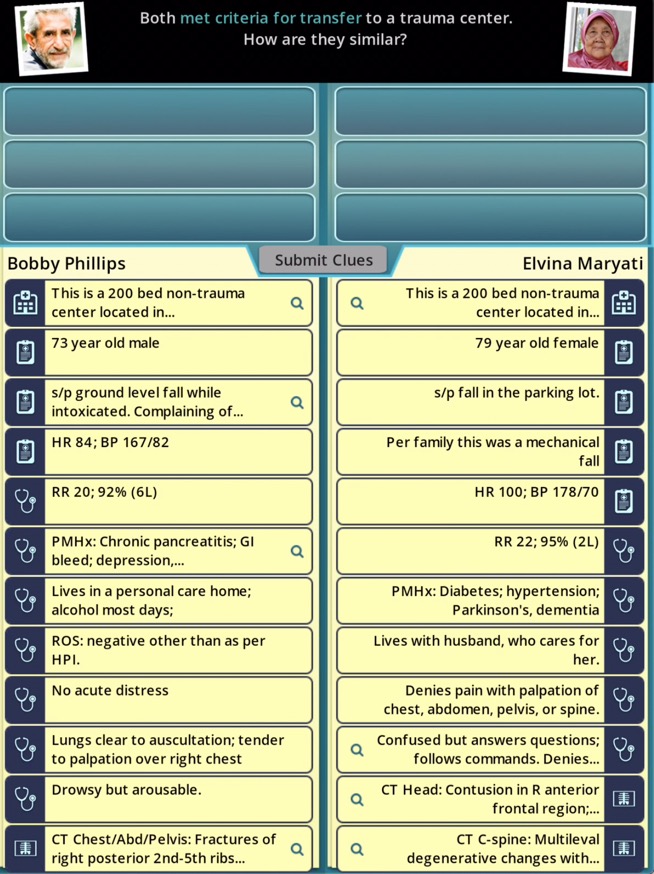 |
| What are predictable errors that might occur? | - Focus on the fact that Phillips is not only old but frail (lives in a personal care home) |
| Case review step | Age + moderate injury --> triage decision |

| Level Ten |  |
| --- | --- |
| Decision Principle | Transfer frail patients with moderate injuries to trauma centers |
| Difficulty | Moderate |
| Bugs | - Evidence of multiple falls or falls at PT (both evidence of frailty in retrospect) were not included in the coding schema. - If you pick the femur fracture for Truong and tibia fracture for Collins, the feedback is that only femur fractures are considered severe - which is correct. But the point of the level is that frailty + moderate injuries --> transfer. So the feedback is slightly incongruent with the teaching point. - There is also a mistake with one of the 'do not transfer' cases - 87 year old Irina Kush with 2 ribs was inappropriately classified as having a minor injury. |
| Case comparison step - the answer is: evidence of frailty (hospitalization 6 months ago [Truong] and wife noticed difficulty walking [Collins]) and moderate injury (bony injury)  Prompt:   - what do you think is most important about this case?   Responses:   - Great. What are other reasons for transfer? - Great. What might have caused that? - Here is some information about what experts think.   Prompt:   - what do you think this means about our triage principle?   Prompt:   - what might keep you from transferring this patient? | 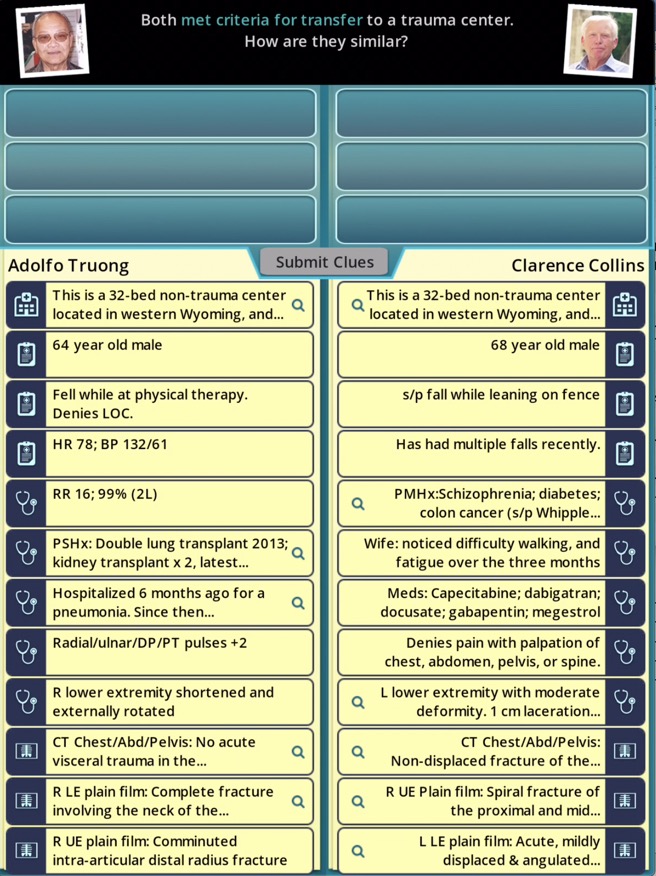 |
| What are predictable errors that might occur? |  |
| Case review step | Frailty + moderate injury --> triage decision |

# Selected References

| American College of Surgeons – Committee on Trauma. (2004) Advanced Trauma Life Support for Doctors: Student Course Manual. Chicago: American College of Surgeons. |
| --- |
| American College of Surgeons. About Advanced Trauma Life Support. <https://www.facs.org/quality-programs/trauma/atls/about>. Accessed: May 29, 2017. |
| Arnold RM, Back AT, Baile WF et al. Oncotalk: Lessons learned. In Kissane D, Bultz B, Butow P, Finlay I. (eds) Handbook of communication in Cancer and Palliative Care. Oxford University Press. 2010. |
| Arogundade RA. Adult learning principles for effective teaching in radiology programmes: a review of the literature. West African Journal of Medicine. 2011; 30: 3-10. |
| Bach JA, Leskovan JJ, Scharschmidt T, et al. The right team at the right time – multi-disciplinary approach to multi-trauma patient with orthopedic injuries. Int J Crit Illn Inj Sci 2017; 7: 32-7. |
| Badjatia N, Carney N, Crocco TJ et al. Guidelines for the prehospital management of traumatic brain injury (2nd edition). Prehospital Emergency Care. 2007; 12: S1-S52 |
| Bellal J, Pandit V, Zangbar B et al. Superiority of frailty over age in predicting outcomes among geriatric trauma patients: a prospective analysis. JAMA Surg 2014; 149: 766-72. |
| Bonne S, and Schuerer DJE. Trauma in the older adult: epidemiology and evolving geriatric trauma principles. Clin Geriatr Med 2013; 29: 137-50. |
| Burns ER, Stevens JA, and Lee R. The direct costs of fatal and non-fatal falls among older adults – United States. *J Safety Res* 2016; 58: 99-103. |
| Calland JF, Ingraham AM, Martin N, et al. Evaluation and management of geriatric trauma: an Eastern Association for the Surgery of Trauma practice management guideline. J Trauma Acute Care Surg. 2012; 73: S345-50. |
| Celso B, Tepas J, Langland-Orban B et al. A systematic review and meta-analysis comparing outcome of severely injured patients treated in trauma centers following the establishment of trauma systems. J Trauma 2006; 60: 371-78. |
| Centers for Disease Control. Injuries cost the US $671 billion in 2013. <https://www.cdc.gov/media/releases/2015/p0930-injury-costs.html>. Accessed May 25, 2017. |
| Chang DC, Bass RR, Cornell EE et al. Undertriage of elderly trauma patients to state-designated trauma centers. Arch Surg 2008; 143: 776-781. |
| Chawda MN, Hildebrand F, Pape HC et al. Predicting outcome after multiple trauma: which scoring system. Injury. 2004; 35: 347-358. |
| Committee on Trauma – American College of Surgeons. Resources for optimal care of the injured patient 2006. Chicago: American College of Surgeons, 2006. |
| Cook A, Weddle J, Baker S et al. A comparison of the Injury Severity Score and the Trauma Mortality Prediction Model. J Trauma 2014; 76: 47-52. |
| Copes WS, Champion H, Sacco, W et al. The injury severity score revisited. J Trauma 1988; 28: 69-77. |
| Delgado, MK, Yokell MA, Staudenmayer KL et al. Factors associated with the disposition of severely injured patients initially seen at non-trauma center emergency departments. *JAMA Surg*. 2014; 149: 422-30. |
| Demetriades D, Karaiskakis M, Velmahos G et al. Effect on outcomes of early intensive management of geriatric trauma patients. Br J Surg 2002; 89: 1319-22. |
| Demetriades D, Martin M, Salim A et al. The effect of trauma center designation and trauma volume on outcome in specific severe injuries. Ann Surg 2005; 242: 512-7. |
| Ericsson A and Pool R. (2017). Peak: secrets from the new science of expertise. Boston, MA: Houghton Mifflin Harcourt. |
| Ericsson KA, Krampe RT, and Tesch-Romer C. The role of deliberate practice in the acquisition of expert performance. Psychol Rev. 1993; 100: 363-406. |
| Ericsson KA. (2018) The differential influence of experience, practice, and deliberate practice on the development of superior individual performance of experts. In The Cambridge handbook of expertise and expert performance. KA Ericsson, A Kozbelt A, and A Mark Williams (Eds.). Boston, MA: Cambridge University Press, pp. 745-769. |
| Ericsson KA. Deliberate practice and the acquisition and maintenance of expert performance in medicine and related domains. Academic Medicine. 2004; 79: S70-S81. |
| Ericsson KA. Expertise and individual differences: the search for the structure and acquisition of experts’ superior performance. WIREs Cogn Sci 2017, 8: e1382. |
| Finkelstein EA, Corso PS, Miller TR et al. The Incidence and Economic Burden of Injuries in the United States. New York: Oxford University Press; 2006. |
| Fischhoff, Baruch. (1982) Debiasing. In Judgment under uncertainty: Heuristics and biases. Daniel Kahneman, Paul Slovic, and Amos Tversky (Eds). New York, NY: Cambridge University Press. |
| Frederick S. Cognitive reflection and decision making. J Econ Persp 2005; 19: 25-42. |
| Fried LP, Tangen CM, Walston J et al. Frailty in older adults: evidence for a phenotype. J Gerontology. 2001; 56: M146-156. |
| Gigerenzer G, Todd PM and the ABC Research Group. Simple heuristics that make us smart. New York: Oxford University Press, 1999. |
| Gomez D, Haas B, de Mestral C, et al. Institutional and provider factors impeding access to trauma center care: an analysis of transfer practices in a regional trauma system. J Trauma. 2012; 73: 1288-93. |
| Goodmanson NW, Rosengart MR, Barnato AE et al. Defining geriatric trauma: when does age make a difference? Surgery. 2012; 668-674. |
| Graber ML, Kissam S, Payne VL, et al. Cognitive interventions to reduce diagnostic error: a narrative review. *BMJ Qual Saf* 2012; 21: 535-557 |
| Haas B, Stukel TA, Gomez D et al. The mortality benefit of direct trauma center transport in a regional trauma system: a population-based analysis. J Trauma 2012; 72: 1510-1517. |
| Hashmi A, Ibrahim-Zada I, Rhee P et al. Predictors of mortality in geriatric trauma patients: a systemic review and meta-analysis. J Trauma 2014; 76: 894-901. |
| HCUPnet 2008. Available at <http://hcupnet.ahrq.gov/HCUPnet.jsp>. Accessed May 20, 2020. |
| Hsia RY, Wang E, Saynina O et al. Factors associated with trauma center use for elderly patients with trauma: a statewide analysis, 1999-2008. Arch Surg. 2011; 146: 585-92. |
| Institute of Medicine. Regionalizing Emergency Care: Workshop Summary. Washington, DC: National Academies Press; 2010. |
| Kahneman D and Frederick S. (2002) Representativeness revisited: attribute substitution in intuitive judgment. In Heuristics and Biases: the Psychology of Intuitive Judgment. Thomas Gilovich, Dale Griffin, and Daniel Kahneman (Eds). New York: Cambridge University Press. |
| Kahneman D and Klein G. Conditions for intuitive expertise: a failure to disagree. *Am Psychol* 2009; 64: 515-526. |
| Kahneman D and Tversky A. (1982) Judgment under uncertainty: Heuristics and biases. In Judgment under uncertainty: Heuristics and biases. Daniel Kahneman, Paul Slovic and Amos Tversky (Eds). New York, NY: Cambridge University Press. |
| Kahneman D. Thinking fast and slow. New York: Farrar, Straus, and Giroux, 2011. |
| Kelley-Quon L, Min L, Morley E et al. Functional status after injury: a longitudinal study of geriatric trauma. *Am Surg* 2010; 76: 1055-1058. |
| Klein G. Sources of Power. Massachusetts: MIT Press, 1985. |
| Kondo Y, Abe T, Kohshi K et al. Revised trauma score system to predict in-hospital mortality in the emergency department: Glasgow Coma Scale, age, and systolic blood pressure score. Crit Care. 2011; 15: R191. |
| Kulkarni K, Dewitt B, Fischhoff B et al. Defining the representativeness heuristic in trauma triage: a retrospective observational cohort study. *PLoS One*. 2019. Forthcoming. |
| Lee L, Patel T, Costa A et al. Screening for frailty in primary care. Can Fam Physician. 2017; 63: e51-e57. |
| Lemov D. (2020). The coach's guide to teaching. Clearfield, FL: John Catt Educational Ltd. |
| Lemov D. (2021). Teach like a champion 3.0. Hoboken, NJ: Jossey-Bass. |
| Lundebjerg NE, Hollmann P, and Malone ML. American Geriatrics Society policy priorities for new administration and 115th Congress. *J Am Geriatr Soc* 2017; 65: 466-469. |
| Macias CA, Rosengart MR, Puyana JC et al. The effects of trauma center care, admission volume, and surgical volume on paralysis after traumatic spinal cord injury. Ann Surg. 2009; 249: 10-17. |
| MacKenzie EJ, Fowler CJ. (2008) Epidemiology. In Trauma. DV Feliciano, KL Mattox, and EE Moore (Eds.). New York: McGraw Medical. Pg. 25-40. |
| MacKenzie EJ, Rivara FP, Jurkovich GJ, et al. A national evaluation of the effect of trauma-center care on mortality. NEJM. 2006; 354: 366-378. |
| MacKenzie EJ, Rivara FP, Jurkovich GJ, et al. The impact of trauma-center care on functional outcomes following major lower-limb trauma. J Bone Joint Surg Am 2008; 90: 101-9. |
| MacKenzie EJ, Weir S, Rivara FP, et al. The Value of Trauma Center Care. *J Trauma* 2010; 69: 1-10. |
| Matsushima K, Schaefer EW, Won EJ et al. Positive and negative volume-outcome relationships in the geriatric trauma population. JAMA Surg. 2014; 149: 319-26. |
| Maxwell CA, Miller RS, Dietric MS et al. The aging of America: a comprehensive look at over 25,000 geriatric trauma admissions to US hospitals. American Surgeon 2015; 81: 630-636. |
| McDonald VS, Thompson KA, Lewis PR et al. Frailty in trauma: a systematic review of the surgical literature for clinical assessment tools. J Trauma 2016; 80: 824-34. |
| McGaghie WC, Issenberg SB, Cohen ER et al. Does simulation-based medical education with deliberate practice yield better results than traditional clinical education? A meta-analytic comparative review of the evidence. Acad Med 2011; 86: 706-711. |
| Miller-Day M and Hecht ML. Narrative means to preventative ends: a narrative engagement framework for designing prevention interventions. Health Comm*.* 2013; 28: 657-670. |
| Mohan D, Angus DC, Ricketts D, et al. Assessing the validity of using serious game technology to analyze physician decision making. PLoS One*.* 2014; 9: e105445. |
| Mohan D, Barnato AE, Angus DC, et al. Determinants of Compliance with Transfer Guidelines for Trauma Patients: A Retrospective Analysis of CT Scans Acquired Prior to Transfer to a Level I Trauma Center. Ann Surg. 2010: 251: 946-51. |
| Mohan D, Barnato AE, Rosengart MR, et al. Trauma triage in the emergency departments of non-trauma centers: an analysis of individual physician caseload on triage patterns. J Trauma. 2013; 74(6): 1541-7. |
| Mohan D, Barnato AE, Rosengart MR, et al. Triage patterns of patients with moderate-to-severe injuries presenting to non-trauma centers. Ann Surg. 2015. 261: 383-389. |
| Mohan D, Barnato AE, Rosengart MR, et al. Triage patterns of patients with moderate-to-severe injuries presenting to non-trauma centers. Ann Surg. 2015. 261: 383-389. |
| Mohan D, Farris C, Fischhoff B, et al. Efficacy of educational video game versus traditional educational apps at improving physician decision making in trauma triage: a randomized clinical trial. BMJ. 2017. 359:j5416 |
| Mohan D**,** Fischhoff B, Angus DC et al. Using serious video games to improve physicians’ heuristics in trauma triage: a randomized clinical trial. PNAS*.* 2018. 115: 9204-9209. |
| Mohan D, Rosengart MR, Farris C, et al. Assessing the feasibility of the American College of Surgeons’ benchmarks for the triage of trauma patients. Arch Surg*.* 2011; 146: 786-792. |
| Mohan D, Rosengart MR, Farris C, et al. Sources of non-compliance with clinical practice guidelines in trauma triage: a decision science study. Implement Sci. 2012; 7: 103. |
| Mohan D, Schell J, and Angus DC. Not thinking clearly? Play a game, seriously! JAMA. 2016; 316(8):1-3. |
| Mohan D, Wallace DJ, Kerti SJ et al. Association of practioner interfacility triage performance with outcomes for severely injured patients with fee-for-service Medicare insurance. JAMA Surgery 2019; 154: e193944. doi: 10.1001/jamasurg.2019.3944 |
| Moore L, Lavoie A, Le Sage N et al. Two worst injuries in different body regions are associated with higher mortality than two worst injuries in the same body region. J Trauma 2006; 60: 802-5. |
| Moran S, Bereby-Meyer Y, and Bazerman M. Stretching the effectiveness of analogical training in negotiations: teaching diverse principles for creating value. Negotiation and Conflict Management Research 2008; 1: 99-134. |
| Morbidity and Mortality Weekly Report. Guidelines for field triage of injured patients: recommendations of the national expert panel of field triage. Atlanta: Department of Health and Human Services – Centers for Disease Control and Prevention, 2009. |
| Nathens AB, Jurkovich GJ, and Maier RV. Relationship between trauma center volumes and outcomes. JAMA 2001; 285: 1164-1171. |
| Nathens AB, Jurkovich GJ, MacKenzie EJ et al. A resource-based assessment of trauma care in the United States. J Trauma. 2004; 56; 173-178. |
| Newgard C, McConnell KJ, Hedges JR. Variability of trauma transfer practices among non-tertiary care hospital emergency departments. Acad Emerg Med 2006; 13: 746-54. |
| Newgard CD, Hedges JR, Adams A et al. Secondary triage: early identification of high-risk trauma patients presenting to non-tertiary hospitals. Prehospital emergency care: official journal of the National Association of EMS Physicians and the National Association of State EMS Directors. 2007; 11: 154-163. |
| Nirula R, Brasel K. Do trauma centers improve functional outcomes: a national trauma databank analysis. J Trauma 2006; 61: 268-71. |
| Prada SI, Salkever D, and Mackenzie EJ. Level 1 trauma center effects on return-to-work outcomes. Eval Rev. 2012; 36: 133-64. |
| Public hearing in the matter of the landing of US Airways Flight 1549, N106US, in the Hudson River, Weehawken New Jersey January 15, 2009. National Transportation Safety Board Office of Administrative Law Judges SA-532. Pages 24-26 (2009) (testimony of Dr Wilson and Captain Sullenberger). |
| Reyna VF and Lloyd FJ. Physician decision making and cardiac risk: effects of knowledge, risk perception, risk tolerance and fuzzy processing. J Exp Psychol 2006; 12: 179-195. |
| Sloman SA. Two systems of reasoning. In Heuristics and biases: the psychology of intuitive judgment. D Griffin, D Kahneman, and T Gilovich (Eds). New York: Cambridge University Press, 2002. |
| Spaite DW, Hu C, Bobrow BJ et al. Mortality and prehospital blood pressure in patients with major traumatic brain injury: implications for the hypotension threshold. JAMA Surg 2017; 152: 360-368. |
| Tillmann BW, Nathens AB, Guttman MP et al. Hospital resources do not predict accuracy of secondary trauma triage: a population-based analysis. |
| US Department of Health and Human Services. Model Trauma System Planning and Evaluation. <https://www.socialtext.net/acs-demo-wiki/index.cgi?regional_trauma_systems_optimal_elements_> integration_and_assessment_systems_consultation_guide. Published February 2006. Accessed November 21, 2010 |
| West TA, Rivara FP, Cummings P et al. Harborview assessment for risk of mortality: an improved measure of injury severity on the basis of ICD-9-CM. J Trauma. 2000; 49: 530-541. |
| Zenati M, Billiar TR, Townsend RN et al. A brief episode of hypotension increases mortality in critically ill trauma patients. J Trauma 53: 232-6. |
| Zhou Q, Rosengart MR, Billiar TR, et al. Factors associated with non-transfer in trauma patients meeting American College of Surgeons’ criteria for transfer at nontertiary centers. JAMA Surg. 2017; 152: 369-376. |

# Consent

Thank you for your interest in this NIH-funded research study. My name is xxxx and I am a researcher at the University of Pittsburgh - School of Medicine. The purpose of this study is to test a deliberate practice intervention for changing physician decision making in trauma. Specifically, we are interested in whether coaching offered in conjunction with a video game would be more effective than a more conventional technique, like ATLS.

As part of this study, we will be randomizing 60 physicians to one of two arms: the intervention or nothing. Physicians in the intervention group will meet with a trauma surgeon virtually for 30 minutes a week, over three weeks, at which time they will play a video game on an iPad that we will provide you, and will receive coaching on key decision principles in trauma triage. They will be interviewed by study personnel after the coaching sessions to provide feedback on their experience, a process that will take about twenty minutes. In addition, physicians in both groups will also complete a virtual simulation online, and fill out two questionnaires. Completing study tasks should take about three hours (unless you are randomized to control arm – in which case you will only need to spend one hour), which you can do at your convenience. We will provide all trial participants with an honorarium of $100/hour spent on study tasks. As per IRS guidelines, all compensation is taxable income to the participant regardless of the amount. If a participant receives $600 or more in a calendar year form one organization, that organization is required by law to file a Form 1099-Miscellaneous with the IRS and provide a copy to the taxpayer. Individuals who do not provide a social security number may still participate in the research, but the IRS requires that 28% of the payment be sent by the institution to the IRS for 'backup withholding;' thus you would only receive 72% of the expected payment.

There will be no direct benefit of participation to you. We will be analyzing the content of your responses to identify how you make decisions for patients in the Emergency Department, and the effect of the intervention on those decisions. The primary risk would be a breach of confidentiality, which might damage your reputation if your description of how you manage patients is judged in a negative fashion. To prevent this from happening, the identifiers will be stored separately, and your responses will be coded and stored on a secure server maintained by the Data Center in the Department of Critical Care Medicine.

The linkage file that ties your name to the identifier will be kept separately, on a secure server behind the University of Pittsburgh firewall. The research team and the University of Pittsburgh Office of Research Conduct and Compliance, will have access to the research records. Also, your research data may be shared with investigators conducting other research; however, this information will be shared in a de-identified manner (without identifiers).

Obviously, your participation in this study is completely voluntary (and much appreciated). You may withdraw at any time. Should you choose to withdraw, all date will continue to be used up to the point of withdrawal unless you request that we destroy it. There is no penalty for refusing to participate or withdrawing. A description of the clinical trial will be available on http://www.clinicaltrials.gov, as required by US Law. This website will not include information that can identify you. At most the website will include a summary of the results. You can search this website at any time. Any questions or concerns should be directed to me as the principal investigator in this study: xxxx. My cell phone number where I can be reached is xxx-xxx-xxxx. If you have any concerns about the study or your rights as a participant, you can contact the University of Pittsburgh human subject protection advocate phone line (1-866-212-2668).

If you are willing to participate, please fill out the following items below which will be used as an electronic signature:

1. Full name
2. Birthdate
3. Name of your high school
4. Are you willing to participate in this study (yes/no)
5. Do you provide your permission to be audio/video recorded for the purposes of this study (yes/no).
